# The Antituberculosis Drug Ethambutol Selectively Blocks Apical Growth in CMN Group Bacteria

**DOI:** 10.1128/mBio.02213-16

**Published:** 2017-02-07

**Authors:** Karin Schubert, Boris Sieger, Fabian Meyer, Giacomo Giacomelli, Kati Böhm, Angela Rieblinger, Laura Lindenthal, Nadja Sachs, Gerhard Wanner, Marc Bramkamp

**Affiliations:** Ludwig-Maximilians-Universität, Fakultät Biologie, Planegg-Martinsried, Germany; Sequella, Inc.

## Abstract

Members of the genus *Mycobacterium* are the most prevalent cause of infectious diseases. Mycobacteria have a complex cell envelope containing a peptidoglycan layer and an additional arabinogalactan polymer to which a mycolic acid bilayer is linked; this complex, multilayered cell wall composition (mAGP) is conserved among all CMN group bacteria. The arabinogalactan and mycolic acid synthesis pathways constitute effective drug targets for tuberculosis treatment. Ethambutol (EMB), a classical antituberculosis drug, inhibits the synthesis of the arabinose polymer. Although EMB acts bacteriostatically, its underlying molecular mechanism remains unclear. Here, we used *Corynebacterium glutamicum* and *Mycobacterium phlei* as model organisms to study the effects of EMB at the single-cell level. Our results demonstrate that EMB specifically blocks apical cell wall synthesis, but not cell division, explaining the bacteriostatic effect of EMB. Furthermore, the data suggest that members of the family *Corynebacterineae* have two dedicated machineries for cell elongation (elongasome) and cytokinesis (divisome).

## IMPORTANCE

Antibiotic treatment of bacterial pathogens has contributed enormously to the increase in human health. Despite the apparent importance of antibiotic treatment of bacterial infections, surprisingly little is known about the molecular functions of antibiotic actions in the bacterial cell. Here, we analyzed the molecular effects of ethambutol, a first-line antibiotic against infections caused by members of the genus *Mycobacterium*. We find that this drug selectively blocks apical cell growth but still allows for effective cytokinesis. As a consequence, cells survive ethambutol treatment and adopt a pneumococcal cell growth mode with cell wall synthesis only at the site of cell division. However, combined treatment of ethambutol and beta-lactam antibiotics acts synergistically and effectively stops cell proliferation.

## INTRODUCTION

Bacterial antibiotic resistance is an increasing problem in human health and has gained tremendous attention in recent years (1). The occurrence of multiple-drug resistance within pathogenic strains has also been described for *Mycobacterium tuberculosis*, the causative agent of tuberculosis (TB) (2). According to a current report by the World Health Organization, the annual death toll due to TB outnumbers the casualties due to HIV infection, indicating that TB is among the most dangerous infectious diseases. An estimated 1.5 million people died from TB infection in 2014, despite the availability of antibiotic treatment. The bacterial cell wall is a prime target for antibiotic treatment, as it is essential for growth and survival. Moreover, in mycobacteria, the cell wall is also required for virulence (3). The cell wall is composed of a complex polymer that can be divided into three distinct layers (4, 5). Mycobacteria contain a classical bacterial peptidoglycan (PG) sacculus, which is covalently connected to an arabinogalactan (AG) layer (5–7). The AG polymer is further linked to long-chain mycolic acids (MAs) (8, 9). The synthesis pathway of this mycolyl-AG-PG (mAGP) complex has been extensively studied over the past years, and many reactions and their corresponding enzymes have been elucidated (4).

*Mycobacterium* species share this complex cell wall composition with other members of the order *Actinomycetales*, such as *Corynebacterium* and *Nocardia* species. This group of bacteria is therefore also known as the *Corynebacterium*, *Mycobacterium*, and *Nocardia* (CMN) group or mycolata (10, 11). Corynebacteria are abundant skin commensals and include notorious pathogens such as* Corynebacterium diphtheriae*. Although the details of the cell wall synthesis pathways are known, the mode of growth and the subcellular assembly of the corresponding molecular machines driving cell wall synthesis are poorly characterized (12). This is surprising in light of the fact that several first-line antibiotics used in TB treatment target steps in cell wall synthesis (13). Ethambutol (EMB) is one of the antibiotics that act specifically against cell wall synthesis in CMN group bacteria (14–17). EMB blocks the polymerization of arabinose subunits in the AG layer of the cell wall (18, 19), which leads to loss of the MA layer. EMB acts bacteriostatically; however, the precise effects of this drug at the single-cell level have not been addressed; how cells react to EMB treatment and how cell growth is affected remain unclear. Here, we used the nonpathogenic model bacteria *Corynebacterium glutamicum* and *Mycobacterium phlei* to unravel the mechanism underlying the action of EMB. *C. glutamicum*, in particular, has emerged as an excellent tool for studying apical growth in bacteria (12).

Previously, we reported on the organization of polar elongation growth in *C. glutamicum* (20, 21). The polar scaffold protein DivIVA (Wag31 in *M. tuberculosis*) serves as the central hub in polar organization through its interaction with the shape, elongation, division, and sporulation (SEDS) protein RodA and the penicillin-binding protein PBP2A, thus providing spatial regulation of the polar elongation complex known as the elongasome (20–23). Recently, it was shown that RodA is a major glycosyltransferase involved in PG synthesis (24). Moreover, DivIVA is involved in the tethering of chromosome origins through an interaction with ParB (25), thereby coupling the cell cycle and growth. However, it is unclear where the CMN group-specific AG and MA layers are added and whether these bacteria contain dedicated machineries for cell wall synthesis at the cell poles and at the division site.

Here, we show that EMB-treated cells specifically arrest cell wall synthesis at the cell poles; i.e., they specifically block elongation growth. However, these EMB-treated cells are still able to form new division septa where new PG is inserted into the cell wall. Although the arabinose content of the cell wall is clearly reduced and the MA layer is lost, these cells survive and switch to pneumococcal-type elongation growth by inserting cell wall material at the division sites. Thus, by maintaining PG synthesis during cell division, the cells remain viable and return to normal growth following the termination of EMB treatment. Our data explain the bacteriostatic effect of EMB and suggest that CMN group bacteria (also termed *Corynebacterineae*) use two distinct machineries for the synthesis of their cell wall, a polar elongasome that synthesizes all three layers of the complex cell wall and a conventional divisome that synthesizes solely PG during cytokinesis.

## RESULTS

### EMB has a bacteriostatic effect on *C. glutamicum* growth.

**FIG 1:**
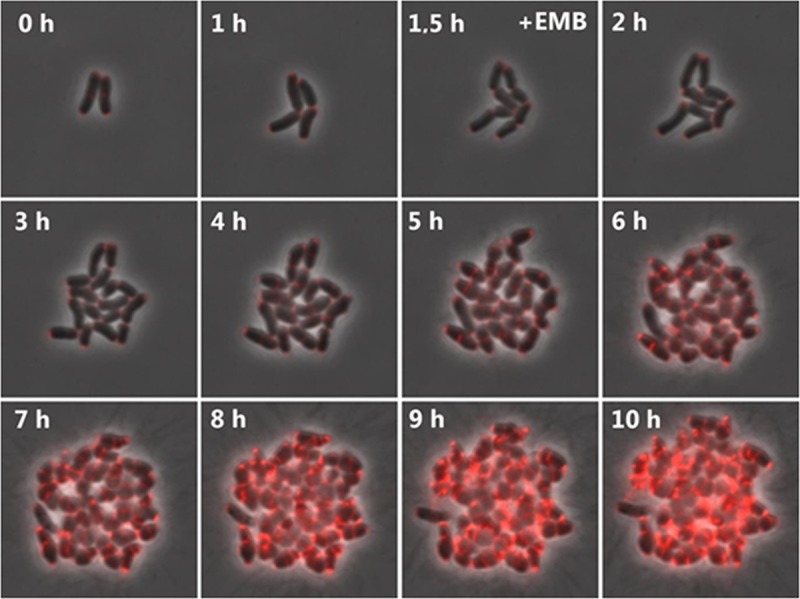
Time-lapse analysis of EMB-treated *C. glutamicum* cells. *C. glutamicum* cells encoding a chromosomal copy of DivIVA-mCherry (red fluorescence) were grown in ready-made microfluidic chambers (CellAsic). Cells were grown in BHI medium for 1.5 h prior to a switch to BHI medium supplemented with EMB (1 mg ⋅ ml^−1^). Upon the addition of EMB, a morphological transition from long, regularly shaped rods to short, roundish cells occurred. Note that at 5 h posttreatment, hydrophobic material appearing as fibers in the phase-contrast images become visible.

EMB is a cell wall-acting antibiotic that targets enzymes that are part of the actinobacterial AG synthesis machinery (19). EMB is most effective against mycobacteria, which require their mAGP layer for viability. In addition to serving as a first-line antibiotic for treating TB, EMB is also the drug of choice for treating various infections caused by nontuberculous mycobacteria (NTM) (17). We chose to use *C. glutamicum* and *M. phlei* as model organisms because of their similar cell wall architecture. First, we tested the effect of EMB on the growth of *C. glutamicum* by using a ready-made microfluidic chamber. To monitor the growth and synthesis of new septa, we used a *C. glutamicum* strain expressing a functional mCherry fusion of the polar growth determinant DivIVA as previously described (25). DivIVA-mCherry localizes to the cell poles and maturing septa by binding to negatively curved membrane regions (25–27). Cells were loaded into the microfluidic chamber, and growth was monitored microscopically. Cells were initially cultured in brain heart infusion (BHI) medium, and once growth commenced (as judged by several rounds of division), the growth medium was changed to BHI including EMB (1 mg ⋅ ml^−1^). Cell growth continued in the presence of EMB; however, the growth rate was decreased and the cells changed from a distinct rod shape to a round, almost coccoidal shape (Fig. 1). An increase in DivIVA-mCherry fluorescence suggests a decrease in elongation growth. DivIVA expression is constitutive during the cell cycle, and the concentration of the DivIVA protein is diluted by cell growth division events. We observed a significant increase in DivIVA protein levels in the cells upon EMB treatment, which appeared to be partly mislocalized (Fig. 1). Starting at 5 h following EMB addition, a hydrophobic material was released from the cell surface, appearing as a fibrous material surrounding the cells. Chemical analysis indicated that these fibers consist of MAs (see Fig. S1 in the supplemental material), which are apparently released from the cell surface. On the basis of these initial findings, we proceeded with an in-depth analysis of the activity of EMB against *C. glutamicum*.

10.1128/mBio.02213-16.1 FIG S1  Detection of MAMEs in the supernatant of EMB-treated cells. (A) Schematic representation of the supernatant analysis. (B) MAMEs were extracted from purified cell walls as methyl esters by acidic methanolysis and analyzed by TLC. We loaded samples of untreated (red box) and EMB-treated (10 µg ⋅ ml^−1^; blue box) cells and a cardiolipin control (black box). The image presented is composed of three technical replicates for each sample; with increasing amounts applied per spot (note the increase in MAMEs in the third lane of the EMB-treated sample). Download FIG S1, TIF file, 0.1 MB. Copyright © 2017 Schubert et al. 2017 Schubert et al. This content is distributed under the terms of the Creative Commons Attribution 4.0 International license.

**FIG 2:**
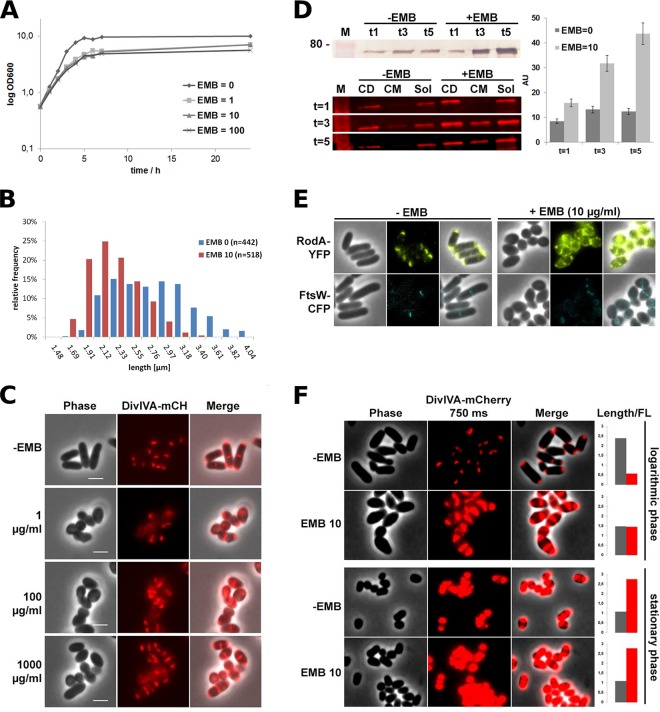
Effect of EMB stress on *C. glutamicum* cells. (A) Growth curves of WT *C. glutamicum* cells treated with different concentrations of EMB. Growth is significantly inhibited by EMB at 1 µg ⋅ ml^−1^. (B) Analysis of cell length with (red bars) or without (blue bars) EMB treatment. (C) Fluorescence microscopy images of *C. glutamicum* DivIVA-mCherry cells (strain BSC003) following treatment with different concentrations of EMB. Morphology appears altered, and cells are shorter and thicker. Moreover, the DivIVA protein level is increased and the protein is mislocalized. Scale bars, 2 µm. (D) Protein quantification following immunoblotting. (Top) Samples of whole-cell extracts from cultures with equal ODs were used for SDS-PAGE and immunoblotting. DivIVA-mCherry was detected with an anti-mCherry antibody and a secondary antibody coupled to alkaline phosphatase. The molecular size marker (M) indicates 80 kDa. (Bottom) DivIVA-mCherry cells were fractionated following lysis by centrifugation and SDS-PAGE. DivIVA-mCherry was detected by in-gel fluorescence. Samples were taken 1, 3, and 5 h after the addition of EMB (*t* = 1 to *t* = 5). Fluorescence quantification of in-gel fluorescence (right diagram) indicates an increase in the DivIVA protein concentration upon EMB addition. Error bars indicate standard deviations. M, marker; CD, cell debris; CM, cell membranes; Sol, soluble fraction; AU, arbitrary units. (E) Fluorescence microscopy of *C. glutamicum* RodA-YFP (strain BSC026) and FtsW-CFP (strain BSC025) following EMB treatment. RodA is mislocalized, and the protein level seems to increase upon the addition of EMB. The FtsW protein level appears to be unaffected, and FtsW localization is restricted to septa. (F) Fluorescence microscopy of DivIVA-mCherry in the exponential and stationary growth phases with or without EMB. EMB targets and affects the polar elongation machinery. Cells in stationary phase that have no distinctive elongation growth are not affected by EMB; morphology and the DivIVA level are not altered.

Growth experiments with BHI medium revealed a decrease in growth rates following the addition of 1 µg ⋅ ml^−1^ EMB, while higher concentrations of EMB did not further increase the retardation of growth (Fig. 2A). The exponential doubling time of wild-type (WT) cells without EMB was 96 min; the addition of EMB increased the doubling time to 138 min. The final optical density at 600 nm (OD_600_) of the shaking cultures was reduced from ~10 without EMB to ~6 with EMB. As predicted and in agreement with the findings of our microfluidic analysis, EMB appears to act bacteriostatically. To determine how many cells survived EMB treatment, we measured CFU counts. Exponential-phase cell cultures with and without EMB were adjusted to an OD of 1, and dilutions were plated on fresh BHI agar plates without EMB (not shown). WT cultures contained 3.7 × 10^8^ CFU; the addition of EMB to growing cells resulted in a reduction to 5 × 10^7^ CFU, suggesting that EMB does not effectively kill *C. glutamicum* cells but rather inhibits growth. Furthermore, we performed live-dead staining of cultures grown in the presence of 0, 10, or 100 µg ⋅ ml^−1^ EMB. Fluorescence microscopy revealed that up to 2% of the cells in untreated cultures were dead. In samples treated with 10 or 100 µg ⋅ ml^−1^ EMB, up to 5% of the cells were dead (Fig. S2). This low percentage of dead cells is in line with the notion that EMB acts bacteriostatically on *C. glutamicum*.

10.1128/mBio.02213-16.2 FIG S2  Live-dead staining of cells during EMB treatment. *C. glutamicum* cells were grown to mid-exponential phase in BHI medium and diluted into medium without EMB, with 10 µg ⋅ ml^−1^ EMB, or with 100 µg ⋅ ml^−1^ EMB. Samples were taken 1 h (A), 2 h (B), and 3 h (C) after EMB treatment, and cells were stained with the Bacteria Live/Dead Staining kit (PromoKine). Growth curves of the cultures are shown in panel D. Fluorescence micrographs are shown for each EMB concentration and time point (live cells appear green, and dead cells appear red). Bar charts represent the statistical analysis of live (gray) and dead (white) cells. For each condition, 1,000 cells were counted (*n* = 1,000). Untreated cell cultures contain 2% dead cells, while cells treated with EMB have 5 to 8% dead cells. Download FIG S2, TIF file, 0.9 MB. Copyright © 2017 Schubert et al. 2017 Schubert et al. This content is distributed under the terms of the Creative Commons Attribution 4.0 International license.

### EMB affects cell morphology.

Next, we analyzed the morphological effects of EMB on *C. glutamicum* cells by using phase-contrast microscopy. Addition of EMB (1 µg ⋅ ml^−1^) to exponentially growing cells resulted in a decrease of approximately 32% in the average cell length, whereas thickness increased to approximately 123%; following EMB treatment, cell length was reduced to 1 ± 0.25 µm, compared to 2 ± 0.25 µm in untreated cells (Fig. 2B). The cell volume was calculated as 1.15 ± 0.64 µm^3^ following EMB treatment, a decrease of approximately 29% compared to that of untreated WT cells (1.62 ± 0.64 µm^3^). Our data are compatible with previous reports regarding morphological changes subsequent to EMB treatment (28).

### EMB treatment causes accumulation of DivIVA.

Having observed morphological alterations such as shorter and thicker cells and reduced growth rates following EMB treatment, we next addressed why EMB-treated cells apparently increase cellular DivIVA levels.

To determine whether DivIVA levels were increased because of upregulated transcription rates, we performed quantitative PCR (qPCR) experiments and compared the DivIVA mRNA level with those of three different housekeeping genes (*thrC* [threonine synthase], *gyrB* [DNA gyrase], and *glnA* [glutamine synthetase]). DivIVA mRNA levels, and thus transcription rates, were not altered upon the addition of EMB (data not shown). A possible explanation for the increased levels is that the constitutive expression of DivIVA leads to accumulation upon growth inhibition, similar to the situation in stationary-phase cells. If transcriptional levels are not altered, it is likely that the protein levels increase over time because of DivIVA accumulation as a result of reduced elongation growth.

Therefore, we next quantified DivIVA-mCherry protein levels. Allelic replacement of *divIVA* at the native locus with an mCherry fusion gene allowed precise protein quantification. Immunoblotting with antibodies directed against the mCherry protein confirmed the increase in DivIVA levels (Fig. 2D, top). As we observed only minor DivIVA degradation products containing the mCherry fusion on the immunoblots and a substantial increase in full-length protein, we concluded that the mislocalization of DivIVA, as seen in the fluorescence micrographs (Fig. 1; Fig. 2C), was due to saturation of the polar regions with DivIVA and a consequent random distribution of DivIVA within the whole cell. A cell fractionation study revealed that the increased concentration of DivIVA-mCherry leads to an enhanced level of membrane-bound DivIVA (Fig. 2D, bottom). In-gel fluorescence measurements of whole-cell extracts (CD), cell membrane fractions (CM), and the cytoplasm (Sol) showed that following 1 to 3 h of EMB treatment, most of the DivIVA is located in the soluble fraction. However, strong enrichment of DivIVA leads to coisolation of DivIVA with the cell membrane, indicating that excess DivIVA might bind to the membrane. The fluorescence quantification results also suggest a substantial increase in DivIVA-mCherry in EMB-treated cells (Fig. 2D, right side). DivIVA-mCherry fluorescence remained unchanged in untreated cells between 1 and 5 h of growth; however, upon EMB treatment, DivIVA-mCherry fluorescence increased >4-fold.

To analyze whether the excess DivIVA found after EMB treatment caused the morphological changes seen, we constructed a strain overexpressing DivIVA (GGB1C9). The plasmid-encoded DivIVA-Dendra2 construct is under the control of an isopropyl-β-d-thiogalactopyranoside (IPTG)-inducible promoter. Overexpression of DivIVA can be monitored by measuring Dendra2 fluorescence. Cells overexpressing DivIVA have a slightly lower growth rate (Fig. S3A) than the WT. Interestingly, cells overexpressing DivIVA are more susceptible to EMB addition. The excess DivIVA-Dendra2 localizes to the poles of untreated cells (Fig. S3B). In untreated cells, DivIVA overexpression causes polar bulging and the morphology of the cells is usually wider with increased cell poles (Fig. S3B). Upon EMB addition, DivIVA accumulates at the cell poles, causing massive shape aberrations. These data suggest that an EMB-caused block of polar growth acts through DivIVA.

10.1128/mBio.02213-16.3 FIG S3  Overexpression of DivIVA renders cells more susceptible to EMB. (A) Shown are growth curves of *C. glutamicum* in the absence (dot-dashed line with triangles) or presence of 10 µg ⋅ ml^−1^ EMB (dotted line with rhomboids). A strain (GGB1C9) overexpressing a functional DivIVA-Dendra2 fusion construct has a lower growth rate than the WT (solid line with squares). Addition of 10 µg ⋅ ml^−1^ EMB to cells of strain GGB1C9 results in a drastic growth reduction (dashed line with circles). (B) Phase-contrast images showing the morphological changes in WT (RES 167) or strain GGB1C9 (DivIVA-Dendra2) cells upon the addition of EMB. Dendra2 fluorescence shows DivIVA-Dendra2 overexpression and the accumulation of excess DivIVA at the cell poles. Download FIG S3, TIF file, 6.8 MB. Copyright © 2017 Schubert et al. 2017 Schubert et al. This content is distributed under the terms of the Creative Commons Attribution 4.0 International license.

### EMB treatment causes mislocalization of the apical growth machinery.

The observed reduction in cell elongation growth points to a block in cell elongation. Although the precise composition of the apical cell growth machinery is unclear, we have previously shown that the SEDS protein RodA is an essential part of the cell elongation machinery (20, 21). Therefore, we used a strain in which the native *rodA* gene was replaced with a *rodA-yfp* fusion gene (21) to study the spatiotemporal localization of the elongation machinery following EMB treatment. In untreated cells, RodA-yellow fluorescent protein (YFP), representing the cell elongation machinery, localized exclusively to the cell poles (Fig. 2E). As a control, we used a strain in which the divisome-specific SEDS protein FtsW was replaced with the FtsW-cyan fluorescent protein (CFP) fusion protein. This fusion is also expressed at its native locus. As predicted, in untreated cells, RodA localizes to cell poles while FtsW localizes to sites of septation. Interestingly, upon EMB addition, RodA-YFP is mislocalized and no longer spatially restricted to the cell poles (Fig. 2E); RodA-YFP localization resembled the dispersed localization of DivIVA in EMB-treated cells. Previously, we have shown that DivIVA recruits RodA to the cell poles via a direct protein-protein interaction (20, 21). Hence, it is possible that EMB treatment induced a block of elongation growth and the resulting mislocalization of DivIVA caused the displacement of the apical growth machinery (elongasome). Consequently, apical growth is reduced and the cells adopt a coccoidal morphology. This mislocalization of RodA is specific; EMB-treated cells still exhibit the typical midcell localization of FtsW-CFP, indicating that the division machinery remains spatially confined and is likely intact.

### EMB treatment results in a stationary-phase-like morphotype.

*C. glutamicum* cells reduce apical growth during the stationary growth phase; the cell morphology of stationary-phase cells is nearly coccoidal. Therefore, we sought to determine whether the cell morphology and DivIVA accumulation levels observed in EMB-treated cells are similar to those of stationary-phase cells. To this end, cells were grown to stationary phase in BHI medium and subsequently treated with EMB. Indeed, we observed a drastic increase in DivIVA-mCherry in stationary-phase cells (Fig. 2F) even in the absence of EMB. Addition of EMB to stationary-phase cells did not alter cell length or the DivIVA-mCherry concentration (as determined by mCherry fluorescence; Fig. 2F). Therefore, we conclude that EMB mimics stationary-phase conditions that lead to increased DivIVA and halt apical elongation growth, hence resulting in morphological changes.

### EMB selectively affects apical cell wall synthesis.

**FIG 3:**
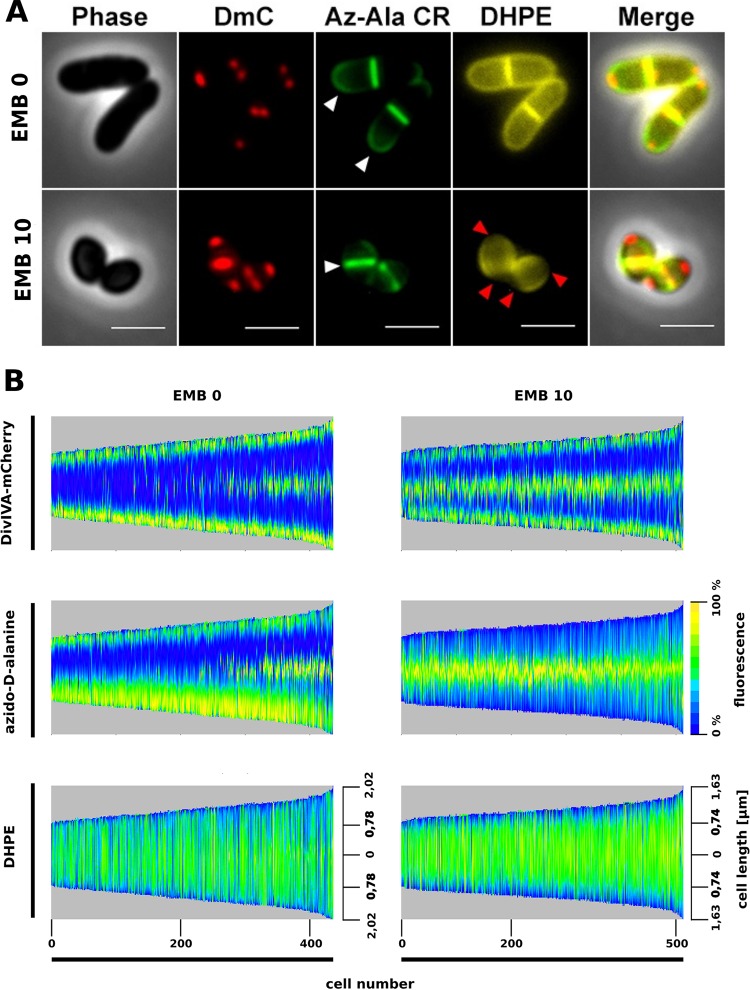
EMB selectively blocks apical cell wall synthesis. (A) Sites of nascent PG in *C. glutamicum* cells (chromosomally encoding DivIVA-mCherry [red]) were labeled by strain-promoted azide-alkyne click chemistry. Azido-d-alanine and DBCO-dye (DBCO-carboxyrhodamine 110 [green]) were added to growing cells (see Materials and Methods) in the absence (EMB 0) or presence of 10 µg ⋅ ml^−1^ EMB (EMB 10). PG synthesis is visible at cell poles and septa (white arrowheads). MA layers were stained with DHPE (yellow). In the presence of EMB, apical PG synthesis is blocked and polar addition of MAs is lost (red arrowheads). (B) Automated image analysis of cell length and fluorescence distribution reveals the spatial localization of PG synthesis and MA distribution in the presence or absence of EMB. The kymographs represent cells sorted by length (shortest cells on the left). Fluorescence intensities are presented as heat maps (blue to yellow). Note the asymmetric fluorescence staining of nascent PG synthesis (azido-d-alanine) at the old cell pole. This coincides with an increased concentration of DivIVA-mCherry at these poles (see Fig. 1 for more quantitative data). EMB causes polar loss of DHPE staining and signal concentration along the side walls (*n* = >400 for EMB 0; *n* = >500 for EMB 10). Movies S1 and S2 are animated GIF videos of all of the cells analyzed demonstrating the localization of DivIVA and PG synthesis.

The observation that EMB inhibition is restricted to exponentially growing cells (Fig. 2F) suggests that EMB only affects the polar cell wall growth machinery. To test whether apical PG synthesis is affected, we utilized a click-labeling approach in which PG-specific d-alanine is replaced with azido-d-alanine, which is subsequently labeled with dyes by click chemistry (29). Cells were grown in the presence of azido-d-alanine, which is incorporated into the pentapeptide subunit of lipid II. Subsequent addition of reactive fluorophores that bind to the azido group allows the visualization of nascent PG. Untreated cells were stained at the poles and septa, as previously demonstrated by vancomycin-FL staining (23, 30), indicating polar cell wall synthesis. A strain expressing DivIVA-mCherry (DmC) was used to show that PG incorporation colocalizes with sites of active PG synthesis (Fig. 3A). Interestingly, azido-d-alanine labeling of EMB-treated cells revealed a complete absence of polar cell wall synthesis. Instead, EMB-treated cells were only stained at their septa (Fig. 3A). This observation indicates that polar PG synthesis is abolished following EMB addition. To obtain more quantitative data, we developed an image analysis tool to automatically analyze fluorescence images. Morpholyzer NX is a Fiji (31)-based plug-in that allows automated measurement of cell length and fluorescence profiles along the cell axis. The cells were then sorted according to their length (shortest cells on the left), and data are presented as kymographs or animated GIF videos (see Movies S1 and S2). Fluorescence intensities measured along the cell axis were plotted as heat maps. Figure 3B shows the results of this automated image analysis for DivIVA-mCherry-expressing *C. glutamicum* cells (left side, cells without EMB treatment). The top, middle, and bottom kymographs represent DivIVA-mCherry localization, azido-d-alanine labeling, and 2-dihexadecanoyl-*sn*-glycero-3-phosphoethanolamine (DHPE) fluorescence distribution (representing MA distribution) (32), respectively. Bio-orthogonal labeling with azido-d-alanine is specific for nascent PG synthesis, while DHPE staining does not discriminate between newly synthesized and old MAs. *C. glutamicum* cells appear to grow asymmetrically with increased PG synthesis at one cell pole (Fig. 3B, middle). Close inspection of the fluorescence images indicated that the old cell pole gives rise to rapid PG synthesis, while synthesis at the young cell pole is slower. The pole with increased PG synthesis also exhibited an elevated DivIVA concentration (top), suggesting that DivIVA assembly is directly linked to cell wall synthesis. Once the cells reached a certain length, a new septum was formed, as indicated by DivIVA and azido-d-alanine staining at the center of the cells. Importantly, new cell wall synthesis and DivIVA overlap at the midcell position, indicating that DivIVA is spatially linked to cell wall synthesis. DHPE staining was dispersed across the entire cell surface with no clear enrichment at the cell poles, indicating that cells are completely surrounded by a MA layer. In comparison with the situation in untreated cells, EMB addition drastically altered the spatial localization of active cell wall synthesis. EMB-treated cells lacked polar cell wall synthesis (Fig. 3B, middle). However, a strong staining pattern was observed surrounding the midcell point at all cell lengths. These data show that cells treated with EMB continue to divide and that cell wall synthesis is completely shifted from the cell poles to the septum. The altered cell wall synthesis state is also reflected by the change in DivIVA localization. In EMB-treated cells, DivIVA localization is reduced at the cell poles and the apparent enrichment of DivIVA at the old cell pole is absent (Fig. S4).

10.1128/mBio.02213-16.4 FIG S4  Statistical analysis of fluorescence localization in *C. glutamicum* in the presence or absence of EMB. The collectives of analyzed cells (EMB 0, *n* = 442; EMB 10, *n* = 518) were divided into five length classes. The bars in each class reflect the relative fluorescence intensity according to the position within the cells. The dark gray bar shows the old cell pole, and the light gray bar shows the new cell pole. The data show the localization of DivIVA-mCherry, the site of PG synthesis labeled with azido-d-alanine, and the MA layer stained with DHPE. Cells treated with EMB (10 µg ⋅ ml^−1^) are shorter and exhibit a lower overall intensity. Download FIG S4, TIF file, 0.7 MB. Copyright © 2017 Schubert et al. 2017 Schubert et al. This content is distributed under the terms of the Creative Commons Attribution 4.0 International license.

10.1128/mBio.02213-16.5 MOVIE S1  Localization of cell wall synthesis and DivIVA in untreated *C. glutamicum*. Shown is an animated GIF video created with Morpholyzer NX. Data were obtained from the kymographs shown in Fig. 3. DivIVA-mCherry localization is red, and azido-d-alanine staining of active cell wall synthesis is green. Shown are the outlines of all of the individual cells that were analyzed for Fig. 3 (EMB 0). Download MOVIE S1, AVI file, 6.6 MB. Copyright © 2017 Schubert et al. 2017 Schubert et al. This content is distributed under the terms of the Creative Commons Attribution 4.0 International license.

10.1128/mBio.02213-16.6 MOVIE S2  Localization of cell wall synthesis and DivIVA in EMB-treated *C. glutamicum*. Shown is an animated GIF video created with Morpholyzer NX. Data were obtained from the kymographs shown in Fig. 3. DivIVA-mCherry localization is red, and azido-d-alanine staining of active cell wall synthesis is green. Shown are the outlines of all of the individual cells that were analyzed for Fig. 3 (EMB 10; 10 µg ⋅ ml^−1^ EMB). Download MOVIE S2, AVI file, 7.3 MB. Copyright © 2017 Schubert et al. 2017 Schubert et al. This content is distributed under the terms of the Creative Commons Attribution 4.0 International license.

### The divisome remains functional in the presence of EMB.

**FIG 4:**
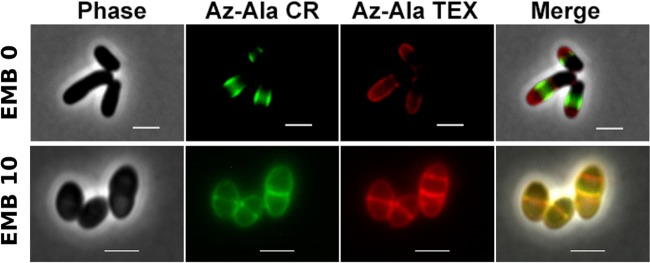
EMB-treated cells synthesize new cell wall at division sites. Shown is pulse-labeling of PG synthesis in *C. glutamicum* cells with azido-d-alanine and DBCO-carboxyrhodamine 110 (green fluorescence) and DBCO-Texas Red (red fluorescence). Cells were pulsed first with DBCO-carboxyrhodamine 110 and then with DBCO-Texas Red. In the absence of EMB (EMB 0, top), cell wall synthesis originates at the cell poles and new PG is transferred to the lateral walls. Note the asymmetric rates of PG synthesis at old and young poles. The young pole is easily identifiable because of the characteristic V-shaped division in corynebacteria. In the presence of EMB (EMB 10, 10 µg ⋅ ml^−1^), cell wall synthesis is blocked at the poles; however, new division sites are initiated, as indicated by septal PG stained exclusively by DBCO-Texas Red (bottom).

Next, we wished to determine whether EMB-treated cells are able to form new septa or whether the observed presence of septal cell wall staining is due to septa initiated prior to EMB treatment. We used a pulse-labeling approach to identify new division events in EMB-treated cells. Cells were first incubated with azido-d-alanine and then subsequently stained with dibenzocyclooctyne (DBCO)-carboxyrhodamine 110 (green). The cells were then further cultivated in the presence of azido-d-alanine prior to the addition of DBCO-Texas Red (red). These pulse-labeling experiments revealed that cells tolerate the incorporation of azido-d-alanine and subsequent click labeling and continue to grow, indicating that azido-d-alanine incorporation is not toxic and does not affect polar growth. Pulse-labeling of untreated cells clearly revealed the asymmetric growth mode of *C. glutamicum*. The old cell pole consistently grew faster and synthesized more cell wall material than the young pole (Fig. 4; Fig. S4). In addition, while there was no apparent polar cell wall synthesis in EMB-treated cells, the formation of new septa was visible. Newly formed septa were observed (red) following a second labeling pulse (Fig. 4). The presence of newly formed septa in EMB-treated cells indicates that the drug does not interfere with divisome function. Hence, the action of EMB appears to be restricted solely to the polar-acting elongasome.

Although corynebacteria and mycobacteria have the same complex cell envelope structure, we wanted to confirm that EMB treatment blocks apical growth in members of the genus *Mycobacterium* in a similar manner. We therefore pulse-labeled cell wall synthesis in *M. phlei* in the presence or absence of EMB (10 µg ⋅ ml^−1^). Similar to *C. glutamicum*, *M. phlei* cells synthesized cell wall PG from their cell poles and septa (Fig. S5A). Upon treatment with EMB, apical cell wall synthesis was entirely blocked in *M. phlei*, indicating that EMB treatment leads to selective blocking of apical growth in mycobacteria as well. Automated image analysis revealed that untreated *M. phlei* cells grow asymmetrically with both rapidly and slowly growing cell poles (Fig. S5B to D). Following the addition of EMB to *M. phlei* cells, polar PG synthesis is lost and new cell wall material is synthesized at the septum, similar to the situation in *Corynebacterium*. Thus, we conclude that EMB treatment leads to the blockage of apical elongation growth in different mycolata members.

10.1128/mBio.02213-16.7 FIG S5  EMB blocks polar cell wall synthesis in *M. phlei*. (A) Pulse-labeling of PG synthesis in *M. phlei* cells with azido-d-alanine and DBCO-carboxyrhodamine 110 (green fluorescence) and DBCO-Texas Red (red fluorescence). The first pulse consisted of DBCO-carboxyrhodamine 110, and the second pulse consisted of DBCO-Texas Red. In the absence of EMB (EMB 0, top), cell wall synthesis originates from the cell poles and new PG is transferred to the lateral walls. Note the asymmetric rates of PG synthesis in old and young poles. In the presence of EMB (EMB 10, 10 µg ⋅ ml^−1^), cell wall synthesis is blocked at the poles but still active at sites of division. (B) Kymograph representations of cells in the absence (EMB 0) or presence (EMB 10) of 10 µg ⋅ ml^−1^ EMB reveal nascent PG synthesis at cell poles and septa (heat map coloring is blue to yellow). Note that PG synthesis in untreated *M. phlei* cells is highly asymmetric, with one fast-growing pole. In the presence of EMB, apical PG synthesis is blocked. (C) Cell length distribution (EMB 0, *n* = 135; EMB 10, *n* = 177). (D) Statistical analysis of fluorescence localization in *M. phlei* in the presence or absence of EMB. The collectives of analyzed cells (EMB 0, *n* = 135; EMB 10, *n* = 177) were divided into five length classes. The bars in each class reflect the relative fluorescence intensity according to the position within the cells. The dark gray bar shows the old cell pole, and the light gray bar shows the new cell pole. The data show the site of PG synthesis labeled with azido-d-alanine. A similar pattern was observed in *C. glutamicum*. Download FIG S5, TIF file, 1.2 MB. Copyright © 2017 Schubert et al. 2017 Schubert et al. This content is distributed under the terms of the Creative Commons Attribution 4.0 International license.

### Superresolution imaging of apical cell wall synthesis in the presence or absence of EMB.

**FIG 5:**
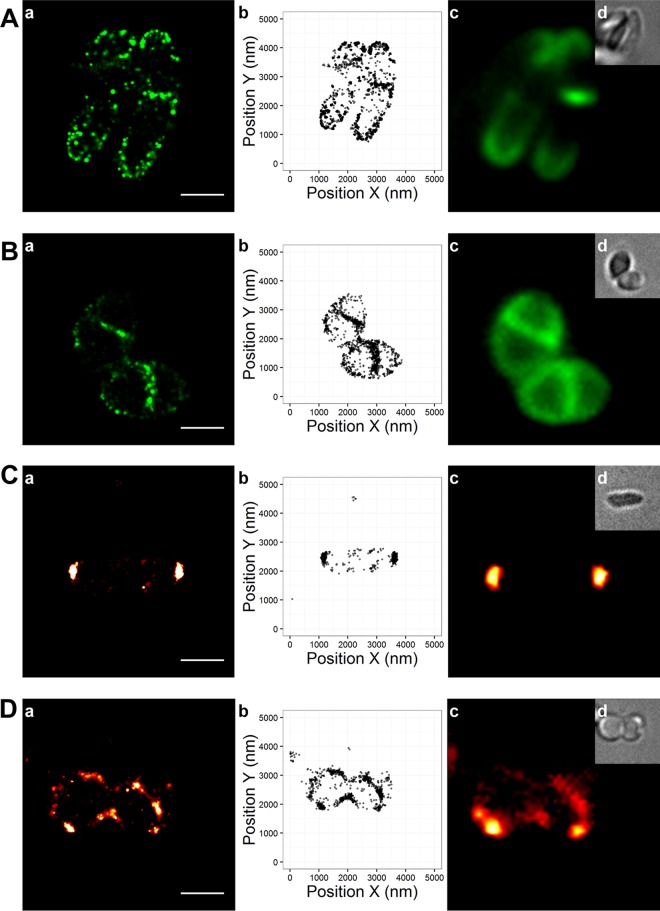
Superresolution imaging of cell wall synthesis and DivIVA localization. Shown are PALM images of PG synthesis in *C. glutamicum* cells with azido-d-alanine stained with DBCO-carboxyrhodamine 110 (A, B) and cells expressing DivIVA-mNeonGreen (strain GGCB1C8, *C. glutamicum* RES 167 *divIVA*::*divIVA-mNeonGreen*) (C, D) grown in the absence (A, C) or presence (B, D) of EMB at 10 µg ⋅ ml^−1^. For each condition, a density rendering (a), a localization plot (b), an epifluorescence image (c), and a bright-field image (d) are shown (scale bar, 500 nm). The newly synthesized cell wall in untreated cells (A) localizes at the poles and at the septum. Similarly, DivIVA-mNeonGreen localizes to cell poles and active septa (C). In EMB-treated cells, PG synthesis is shifted from the cell poles to the septum, suggesting a lack of or diminished polar growth (B). EMB-treated cells exhibit partial loss of polarity, leading to DivIVA delocalization around the membrane (D).

Conventional wide-field imaging is diffraction limited, and hence, spatial localization of proteins can only be estimated within a 250-nm range. We therefore used photoactivated localization microscopy (PALM) to visualize the spatial distribution of DivIVA and nascent PG synthesis with subdiffraction precision. The DBCO-carboxyrhodamine 110 dye used for analysis of nascent cell wall synthesis possesses blinking properties that are essential for localization microscopy (exact parameters are provided in Materials and Methods). Cells with or without EMB treatment were grown in the presence of azido-d-alanine and subsequently labeled with DBCO-carboxyrhodamine 110. The cells were then fixed and imaged with a 488-nm laser line. Nascent PG incorporation into exponentially growing cells without EMB was found to localize (e.g., DBCO-carboxyrhodamine 110 localization) primarily at the extreme cell poles (Fig. 5A). Additional cell wall synthesis was concentrated at the site of septation. In contrast, in EMB-treated cells, polar PG synthesis was nearly abolished, while septal PG synthesis was still present (Fig. 5B). The sites of active cell wall synthesis coincide with DivIVA localization; therefore, we analyzed DivIVA localization by superresolution microscopy. To do so, we first constructed a strain carrying a d*ivIVA-mNeonGreen* fusion gene at the native *divIVA* locus. The resulting strain (GGCB1C8) produces only DivIVA-mNeonGreen. Growth, cell length, and DNA segregation were unaffected in strain GGCB1C8, indicating that DivIVA-mNeonGreen is fully functional (data not shown). Localization microscopy was performed with the 488-nm laser, and as expected, DivIVA-mNeonGreen localized precisely to the cell poles and sites of septation in untreated cells (Fig. 5C). Following EMB treatment, DivIVA localization is dispersed into smaller foci and patches along the cell membrane without a clear polarity axis (Fig. 5D), supporting the data obtained by wide-field microscopy. These data indicate that *C. glutamicum* cells grow through the insertion of PG material at the extreme cell poles. Importantly, this process is abolished following EMB treatment. Although DivIVA localization is dispersed in EMB-treated cells, cell wall synthesis at the sites of division continues to occur with high precision, indicating that the activity and placement of the divisome are unaffected by EMB treatment.

### EMB-treated cells have a compromised outer cell envelope.

**FIG 6:**
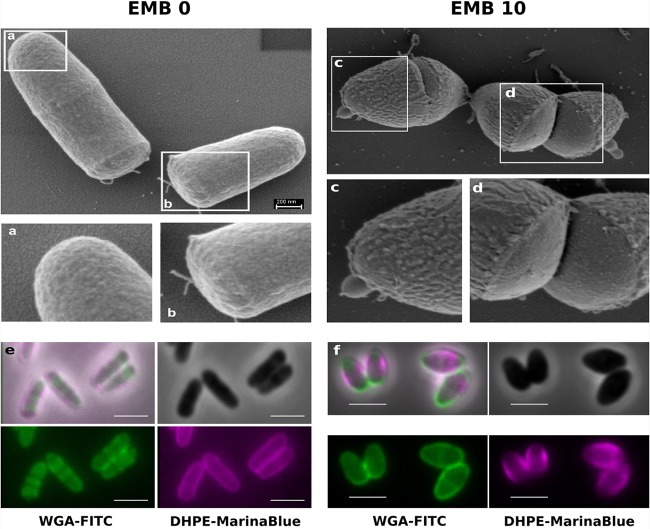
Loss of the outer cell envelope layer in *C. glutamicum* following EMB treatment. SEM images of untreated *C. glutamicum* cells (EMB 0) reveal a smooth, continuous cell surface at old (a) and young (b) cell poles, while cells treated with EMB (EMB 10, 10 µg ⋅ ml^−1^) show a rough cell surface at the lateral sites and the old cell poles (c). Cells actively divide in the presence of EMB (d) and display a smooth, homogeneous surface. (e) Staining with WGA-FITC (green) and DHPE-MarinaBlue (purple) reveals weak uniform WGA staining in untreated cells. (f) In EMB-treated cells, WGA-FITC fluorescence is enhanced at the cell poles and septa, indicating increased binding of WGA-FITC to *N*-acetyl-d-glucosamine. These sites coincide with the lack of the MA layer as determined by the absence of DHPE staining, suggesting that PG constitutes the outer layer of the cell wall at dividing septa following EMB treatment.

The apparent cell wall synthetic activity of the divisome in the presence of EMB suggests that the cell wall synthesized by the division machinery might not contain AG layers and, hence, not be sensitive to EMB. Rather, the divisome might only synthesize PG, as observed in other bacteria. To obtain a more detailed understanding of the surface structure of cells in the presence or absence of EMB, we used scanning electron microscopy (SEM) imaging. *C. glutamicum* cells grown in BHI without EMB exhibit a smooth homogeneous surface (Fig. 6). A magnified view of the poles of freshly divided daughter cells (Fig. 6a and b) revealed a homogeneous cell surface at both the old (Fig. 6a) and new (Fig. 6b) cell poles. In contrast, EMB-treated cells had a rough cell surface with uneven distribution of outer material (Fig. 6c). Interestingly, the cell surface at the division sites seemed rather smooth, indicating that the rough outer layer was no longer present at the new division sites once the cells were treated with EMB (Fig. 6d). We hypothesized that the material synthesized at the septum is PG without the mycolata-specific AG and MA envelope layers. To test this hypothesis, we stained untreated and EMB-treated cells with wheat germ agglutinin (WGA). WGA is a wheat lectin that binds to *N*-acetyl-d-glucosamine. For fluorescence imaging, the WGA was labeled with fluorescein isothiocyanate (FITC). When untreated cells were incubated with WGA-FITC, only a diffuse, weak fluorescence signal was observed surrounding the entire cell surface (Fig. 6e). This is likely due to the existence of a complete MA layer surrounding the cells. DHPE-MarinaBlue labeling uniformly stained the entire cell surface, demonstrating that the MA layer is shielding the entire cell surface. The DHPE-MarinaBlue staining patterns in EMB-treated cells indicate that cell pole areas are devoid of (or have a reduced amount of) MAs. WGA-FITC selectively labels the regions were the DHPE-MarinaBlue stain is absent (Fig. 6f). These findings are in agreement with the hypothesis that the cell wall material synthesized by the divisome consists primarily of PG, identified by the presence of *N*-acetyl-d-glucosamine, which is surface exposed in the presence of EMB. To further corroborate the idea that PG is more exposed in cells treated with EMB, we analyzed the susceptibility to lysozyme, an *N*-acetylmuramide glycanhydrolase known to degrade the sugar backbone of PG. Indeed, cells treated with EMB are more sensitive to lysozyme treatment than untreated cells are (Fig. S6).

10.1128/mBio.02213-16.8 FIG S6  Influence of lysozyme on EMB-treated cells. Shown are growth curves of WT cells treated with lysozyme (solid line with filled squares) in comparison with that in untreated cells (dotted line with triangles) and cells treated with lysozyme and 10 µg ⋅ ml^−1^ EMB. Note that addition of EMB renders the cells more sensitive to lysozyme. Download FIG S6, TIF file, 3.2 MB. Copyright © 2017 Schubert et al. 2017 Schubert et al. This content is distributed under the terms of the Creative Commons Attribution 4.0 International license.

### EMB alters the composition of the cell wall.

**FIG 7:**
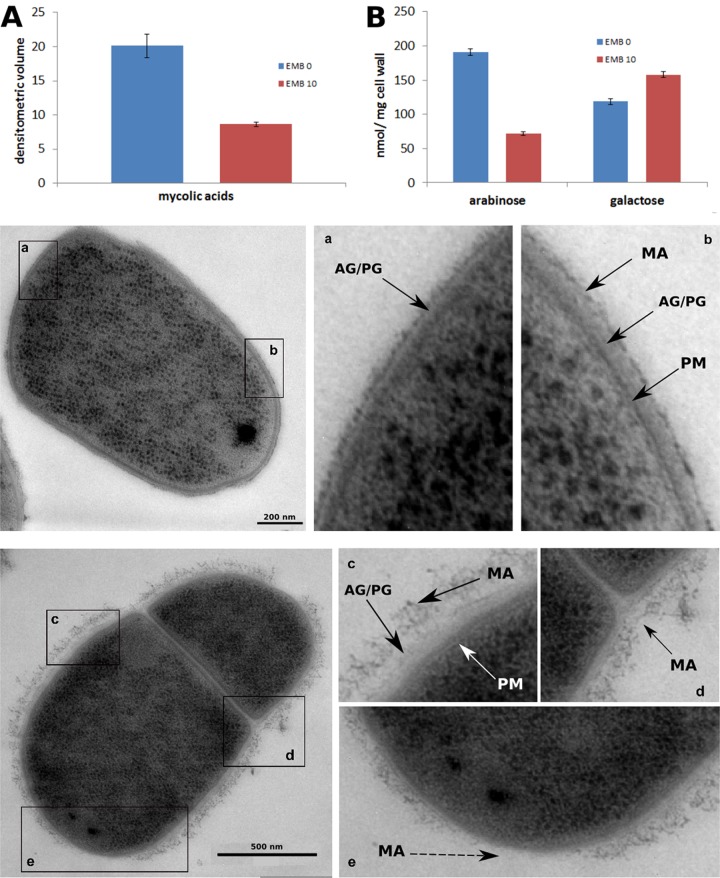
Loss of arabinose and MAs from the cell envelope of EMB-treated cells. (A) Analysis of the MA content of EMB-treated cells (red column) and untreated control cells (blue column). Addition of EMB (10 µg ⋅ ml^−1^) leads to a drastic reduction of the MA content. MAMEs were quantified by TLC (see Materials and Methods). (B) TLC analysis of AG sugars in the presence or absence of EMB. Sugars were quantified in nanomoles per milligram of cell wall material. Note the specific reduction of arabinose following EMB treatment. Transmission electron microscopy of untreated *C. glutamicum* cells reveals the multilayered cell envelope of CMN group bacteria. AG/PG, AG/PG cell wall; MA, MA layer. Untreated cells have a smooth and even MA layer surrounding the cells (a, b). EMB-treated cells exhibit a rough MA layer (c) that is selectively lost from cell poles (e).

EMB inhibits the arabinosyltransferases EmbABC (*C. glutamicum* only encodes EmbC), thereby blocking polymerization of the arabinose chains in the AG layer (18, 19). Terminal arabinose subunits are covalently linked to MAs. Consequently, loss of arabinose polymers will result in reduced levels of MA (33, 34). We therefore sought to determine the direct effects of EMB treatment on cell wall composition. AG extracted from cells with and without EMB treatment was hydrolyzed, and the resulting sugars were separated by thin-layer chromatography (TLC) (Fig. 7A; Fig. S7). This analysis revealed that the arabinose content of the EMB-treated cells was significantly reduced to 10 to 20%, confirming the report that EMB targets arabinan synthesis (19). The galactose content was unchanged (note that we identified 16% more galactose in the cell walls isolated after EMB treatment), suggesting the presence of an intact galactan polymer (Fig. 7B). It is likely that the galactan polymer is still covalently bound and thus remains part of the complex cell wall sacculus.

10.1128/mBio.02213-16.9 FIG S7  TLC analysis of cell wall components following EMB treatment. (A) MAMEs. MAs were extracted from purified cell walls as methyl esters by acidic methanolysis and analyzed by TLC. TLC plates were run in toluene-acetone (97:3, vol/vol) and developed with molybdatophosphoric acid. The image presented is composed of three technical replicates for each sample; an extract from 1 mg of cell wall was applied per spot. (B) AG constituents. AG was released from purified cell walls by mild sulfuric acid hydrolysis, and the glycosidic bonds were cleaved with hydrochloric acid. Sugar hydrolysates were run on TLC in acetonitrile-water (85:15, vol/vol) and visualized with aniline/diphenylamine reagent. The image presented is composed of three technical replicates for each sample; hydrolysates corresponding to 0.2 mg of cell wall were applied per spot. EMB 0, untreated control; EMB 10, culture treated with 10 µg ⋅ ml^−1^ EMB. Sugars are indicated on the right. GlcN, glucosamine; Gal, galactose; Ara, arabinose; Rha, rhamnose. Download FIG S7, TIF file, 9.4 MB. Copyright © 2017 Schubert et al. 2017 Schubert et al. This content is distributed under the terms of the Creative Commons Attribution 4.0 International license.

To investigate whether alterations to cell wall composition have direct effects on cell wall structure, we analyzed untreated and EMB-treated *C. glutamicum* cells by transmission electron microscopy (Fig. 7). Untreated cells demonstrated a smooth and even cell surface (Fig. 7a and b), confirming the surface structure observed in the SEM images. Close inspection of the SEM images allowed the identification of the plasma membrane (PM), the cell wall composed of PG and AG, and the outer layer of MAs. In comparison, cells treated with EMB show a dispersed and frayed outer layer, likely indicating the loss of MA layers (Fig. 7c to e). This observation agrees with the rough outer layer observed in SEM images (Fig. 6) and the loss of hydrophobic material during time-lapse imaging (Fig. 1; Fig. S1). Importantly, the selective loss of the outer cell envelope layers from the cell poles (Fig. 7d and e, broken arrow) indicates that the cell poles are the first region to lose MA following the addition of EMB. These findings support the DHPE staining results and indicate that the MA layer is assembled at the poles.

### EMB and β-lactams act synergistically on *Corynebacterium*.

**FIG 8:**
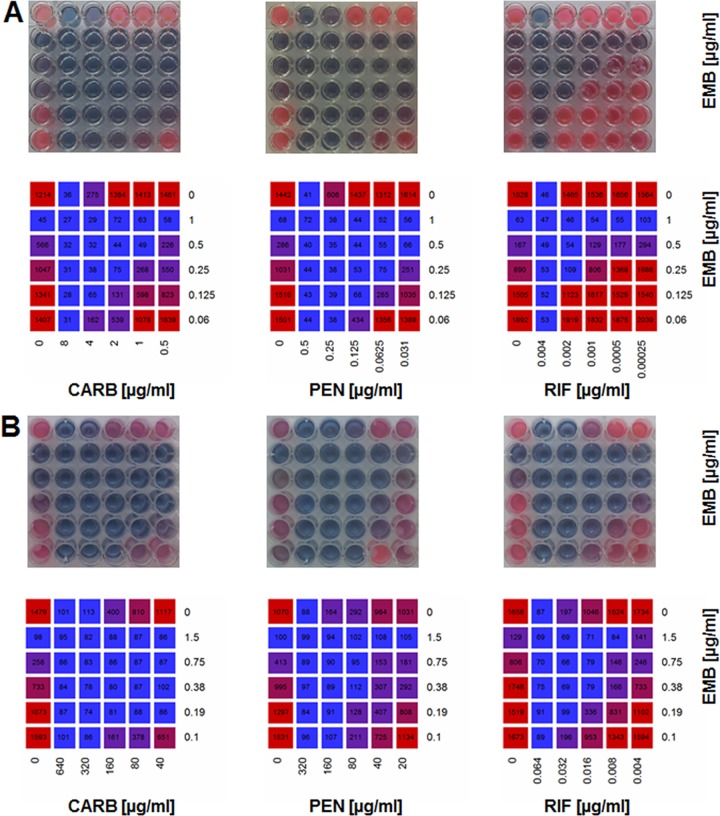
Synergistic effects of EMB and β-lactam antibiotics detected with checkerboard REMA. *C. glutamicum* (A) and *M. phlei* (B) were grown in microtiter plates with combinations of two antibiotics (added at 1:2 dilutions of the MIC). The first horizontal and vertical rows received only one antibiotic and served as the controls. Antibiotic concentrations are shown in micrograms per milliliter. Growth was detected by checkerboard REMA. Heat maps of resorufin fluorescence values were prepared in R (mean values from two to five experiments). (Top) REMA of a typical experiment. (Bottom) Heat maps of fluorescence values.

Our data suggest that EMB treatment selectively blocks apical cell growth and leads to loss of arabinose and MAs from the cell envelope. However, cell division still occurs and the cells divide with an apparently intact PG layer. Thus, we hypothesized that EMB treatment might have synergistic effects with other antibiotics acting on PG synthesis. Therefore, we first determined the MICs of various antibiotics, such as penicillin G (PEN), carbenicillin (CARB), rifampin (RIF), and EMB. To assess the putative additive or synergistic effects, we used a checkerboard assay to study combinations of PEN, CARB, and RIF with EMB. The RNA polymerase inhibitor RIF was used as a control because it does not target cell wall synthesis. Furthermore, RIF is commonly used in TB treatment. Fractional inhibitory concentrations (FICs) and FIC indices (∑FICs) were calculated according to Lechartier et al. (35). As expected, the combination of RIF and EMB had an additive effect in the case of *C. glutamicum* (∑FIC of 0.75). The combination of EMB with the β-lactam drugs PEN and CARB, however, showed a substantial increase in sensitivity. In the presence of EMB, *C. glutamicum* has a ∑FIC of 0.5 for CARB and 0.375 for PEN (Fig. 8A; Table 1). These results suggest that combinations of β-lactams with EMB act synergistically (∑FIC of ≤0.5). Importantly, similar results were obtained with *M. phlei* (Fig. 8B; Table 2), indicating that the synergistic effects of β-lactams and EMB occur in general in *Corynebacterineae*. It should be noted, however, that in this assay also the combination of RIF and EMB had synergistic effects. The reason could be that the high concentrations of β-lactams necessary to reach the MIC lead to pleiotropic effects on the cells. As already shown by increased sensitivity to lysozyme, these data once more support the hypothesis that cells depleted of their MA layer and arabinose polymer sheet have exposed cell wall PG, making them more sensitive to β-lactam treatment.

**TABLE 1:** Antibiotic sensitivity of *C. glutamicum* in the absence of EMB and interaction profile with a second antibiotic

Antibiotic	MIC (µg/ml)	Interaction profile with EMB	
		ΣFIC	Outcome
PEN	0.5	0.375	Synergistic
CARB	8	0.5	Synergistic
RIF	0.004	0.75	Additive

**TABLE 2:** Antibiotic sensitivity of *M. phlei* in the absence of EMB and interaction profile with a second antibiotic

Antibiotic	MIC (µg/ml)	Interaction profile with EMB	
		ΣFIC	Outcome
PEN	320	0.5	Synergistic
CARB	640	0.25	Synergistic
RIF	0.064	0.5	Synergistic

## DISCUSSION

Apical cell growth is a hallmark of bacteria belonging to the phylum *Actinobacteria* (12, 36). However, it is still unclear how this apical growth machinery is assembled and how it might differ from the machinery that synthesizes cell wall material during cell division. In classical model organisms such as *Escherichia coli* and *Bacillus subtilis*, two dedicated machineries are responsible for elongation growth (elongasome) and division (divisome) (37). In *Actinobacteria*, the situation is less clear, although accumulating evidence points to the existence of a dedicated elongasome and divisome (21, 27). *Corynebacterineae* is a suborder within the phylum *Actinobacteria* that includes notorious pathogens such as *M. tuberculosis* and *C. diphtheriae*. A unique feature of bacteria in this suborder is that the mAGP complex constitutes the largest structure in the cell envelope (4, 5). mAGP integrity is essential for *M. tuberculosis* viability and infection. However, it can be partially deleted from *C. glutamicum*. Therefore, *C. glutamicum* is an ideal model system in which to study the influence of anti-TB drugs and their precise mechanism on a single-cell level. Here, we have addressed the effect of EMB on cell growth and morphology. EMB has been previously shown to affect the cell envelope most likely by inhibiting the polymerization of cell wall arabinan (14, 18, 19, 28, 34). Strains resistant to EMB often carry mutations in the arabinosyl transferases EmbABC (18, 19). Deletion of the EMB-targeted arabinosyl transferase EmbC from* C. glutamicum* leads to a truncated AG layer lacking the arabinose polymers and cell wall-bound corynomycolates (34). Treatment with EMB was shown to mimic these phenotypes (34). The loss of apical mycolates has been demonstrated with the MA-specific dye DHPE, suggesting that the mycolate layer is added at the cell poles (32). Our data extend these investigations and show that EMB treatment specifically blocks elongation growth. This was demonstrated by a click-labeling approach to localize sites of active cell wall synthesis by azido-d-alanine staining. Whereas untreated *C. glutamicum* and *M. phlei* cells were stained at the poles and septa, EMB-treated cells were stained exclusively at the septa. Interestingly, PG synthesis at the sites of division was not affected by EMB. Using pulse-labeling experiments in which growing cells were labeled with azido-d-alanine and subsequently modified with two fluorescent dyes, we could show that PG synthesis at the septum continues during EMB treatment and leads to the formation of viable coccoidal cells that have a PG cell wall but lack most of the mycolyl-AG layer. These data are consistent with the hypothesis that *C. glutamicum* and *M. phlei* have a distinct elongasome that is exclusively inhibited by EMB and a divisome that is EMB insensitive; this explains the bacteriostatic nature of EMB. Importantly, we could show that the block in elongation growth caused by EMB mimics reduced cell elongation during the stationary growth phase. We used DivIVA-mCherry accumulation as an easy-to-follow marker of cell elongation and division (25). DivIVA is constitutively expressed, and addition of EMB does not alter the transcription of the *divIVA* gene. Under normal growth conditions, the level of DivIVA is diluted by growth and division. However, in stationary-phase cells or following cell elongation blockage by EMB, DivIVA accumulates and localizes randomly within the cells. DivIVA has been previously demonstrated to be a key spatial organizer of the apical growth machinery via the recruitment of RodA (20, 21) and mAGP complex synthesis proteins (27). In EMB-treated cells, RodA is dispersed throughout the cells. The apparent mislocalization (or perhaps disassembly) of the apical growth machinery explains the block of elongation growth. In contrast, proteins of the divisome remain unchanged following EMB treatment, as indicated by the localization of FtsW. Our data indicate that PG production is continued by the cell division machinery in a coccoid-type manner. These data have two important implications. It is tempting to speculate (i) that the elongasome synthesizes all three cell wall layers (mAGP) in a large supercomplex while the divisome synthesizes only PG and (ii) that treatment with EMB forces cells into stationary growth phase behavior. In consequence, stationary-phase cells are not affected by EMB, as judged by morphology or DivIVA-mCherry fluorescence. The latter is an important factor when considering antibiotic treatment regimens. In most cases, the best clearance of the bacterial load is achieved when cells are actively dividing. *M. tuberculosis*, in particular, is notorious for its long dormancy periods during which the bacteria are difficult to treat (38). Elongation growth appears to be nonessential for *C. glutamicum* viability. When polar elongation is blocked by EMB (or during stationary growth), the division machinery is capable of providing PG synthesis that is sufficient for viability. CMN group bacteria exhibit a remarkable plasticity in response to drug treatment that allows the adaption of these bacteria to survive antibiotic treatment directed against the mAGP complex.

Pulse-labeling of growing cells with azido-d-alanine clearly revealed the asymmetric growth of *C. glutamicum*. In all cells, the old pole synthesized consistently more PG than the young pole. A simple explanation for this is that the elongation machinery needs to assemble after cytokinesis and therefore the young pole lags behind; this young cell pole then slowly matures until it reaches full synthesis capacity. We found that DivIVA concentrations are higher at the fast-growing pole, in line with the role of DivIVA as a scaffold for cell wall synthetic complexes. Following division, the new cell poles are in a rebuilding phase in which the divisome is disassembled and an elongasome is built. This phase leads to slowed elongation growth at the young pole until the elongasome is fully assembled. Asymmetry in growth has been reported previously for *M. tuberculosis* and *M. smegmatis* (39, 40). This growth asymmetry, or more precisely the inheritance of old poles, has been shown to influence antibiotic susceptibility (39). However, this observation has been recently challenged (41); it has been suggested that the difference in polar growth between the old and young poles is due not to pole age but rather to the larger size of the old pole (42). Our data clearly show that inheritance plays a role in the polar growth of *C. glutamicum*. We could show that the old pole usually has a higher level of accumulated DivIVA protein, which in turn might recruit more cell wall synthesis complexes to the cell pole. DivIVA accumulation is a time-dependent process, and hence, the old poles have established a more active elongasome than the young poles.

It has recently been proposed that cell wall synthesis in mycobacteria does not occur precisely at the pole tip but rather is spatially confined to subpolar regions (27). Similar cell wall labeling experiments in this study revealed clear polar growth in *C. glutamicum* cells by wide-field microscopy (Fig. 3 to 4). Since wide-field microscopy is diffraction limited, we used localization microscopy to address the spatial localization of nascent PG synthesis (visualized by azido-d-alanine staining) and DivIVA localization. The DBCO-carboxyrhodamine 110 dye has blinking properties that can be exploited for localization microscopy. Imaging data with a resolution of approximately 50 nm in the *x-y* (the peak localization precision is 30 nm for DBCO-carboxyrhodamine 110 and 25 nm for DivIVA-mNeonGreen) direction indicate a clear enrichment of cell wall synthesis at the extreme cell pole. This coincides with the region of DivIVA localization, as shown by PALM imaging with DivIVA-mNeonGreen. Thus, in *Corynebacterium*, cell wall synthesis appears to be polar and not subpolar, as previously reported for mycobacteria (27). PALM imaging confirms the loss of apical cell wall synthesis and the mislocalization of DivIVA following EMB treatment. Apical growth (in comparison with subapical cell wall synthesis zones) is compatible with previous findings showing a direct protein-protein interaction between DivIVA and RodA. RodA was supposed to act as a lipid II flippase and may also regulate the activity of its cognate penicillin-binding protein (20, 21). However, recent data suggest that RodA rather is a glycosyltransferase involved in PG polymerization (24). It is currently unclear whether SEDS proteins such as RodA and FtsW may act as flippases and glycosyltransferases. The tight interaction between RodA and DivIVA can also be inferred from the simultaneous mislocalization of both proteins upon the addition of EMB. The EMB-caused blockage of cell elongation and the associated mislocalization of excess DivIVA result in the recruitment of RodA by DivIVA to artificial sites within the cell. Overexpression of DivIVA renders the cells more susceptible to EMB, suggesting that the EMB-induced block of apical growth acts through DivIVA. However, at present, we are unable to unambiguously distinguish whether RodA and DivIVA mislocalization cause the blockage in elongation growth or are a direct consequence thereof.

The emergence of multidrug-resistant (MDR) and extremely drug resistant *M. tuberculosis* strains has become a serious health threat and has initiated the search for new therapeutic strategies. These include revisiting the use of β-lactam antibiotics (43, 44). *M. tuberculosis* is resistant to most β-lactams because of its impermeable outer membrane; its potent, chromosomally encoded β-lactamases; and the fact that a large proportion of the cell wall PG is l,d cross-linked, a cell wall linkage that is insensitive to β-lactam antibiotics. However, combinatorial treatment with the β-lactamase inhibitor clavulanic acid and carbapenems has been shown to effectively decrease the bacterial load in mouse models (45). The synergistic effects of β-lactams in combination with clavulanic acid and EMB on MDR strains have been previously shown (46–48). However, within these studies, the underlying molecular reasons for the synergistic effect of EMB and β-lactams remained unclear. Here, we show that EMB effectively removes the outer layers of the mAGP complex and that this, by blocking elongation growth, prohibits new synthesis of these outer envelope layers. As cytokinesis seems to be virtually unaffected by EMB, cell division creates a cell wall composed predominantly of exposed PG, which is hence highly accessible to β-lactam antibiotics. This notion is supported by the increased lysozyme sensitivity of EMB-treated cells. Thus, simultaneous treatment with EMB and β-lactams does allow for the efficient elimination of bacterial cells.

The results described here contribute to a more detailed understanding of elongation growth in CMN group bacteria and aid in our understanding of the molecular action of EMB treatment. This adds to the ongoing and increased effort to improve therapeutic procedures to cure TB or emerging NTM infections and may help to find suitable synergistic antibiotic combinations for effective treatments.

## MATERIALS AND METHODS

### Bacterial strains and cultivation.

The strains used in this study are listed in Table S1. *C. glutamicum* strains were grown in BHI medium (Oxoid). *M. phlei* was cultivated in DSMZ medium no. 219 (yeast extract, 2.0 g; tryptically digested casein peptone, 2.0 g; Na_2_HPO_4_ ⋅ 12H_2_O, 2.5 g; KH_2_PO_4_, 1.0 g; Na citrate, 1.5 g; MgSO_4_ ⋅ 7H_2_O, 0.6 g; glycerol, 50 ml; Tween 80, 0.5 ml/liter [pH 7.0]). Prior to inoculation, 1‰ silicon oil (a 1% suspension in water added to reduce surface tension and thus the clumping of cells) was added to the medium. EMB was dissolved in water and added from stock solutions.

10.1128/mBio.02213-16.10 TABLE S1  Strains, plasmids, and primers used in this study. Download TABLE S1, DOCX file, 0.02 MB. Copyright © 2017 Schubert et al. 2017 Schubert et al. This content is distributed under the terms of the Creative Commons Attribution 4.0 International license.

### Construction of *C. glutamicum* RES 167 *divIVA*::*divIVA*-mNeonGreen.

To generate plasmid pk19mobsacB-divIVA-mNeonGreen-KI, which allows allelic replacement of *divIVA* with a *divIVA* C-terminally tagged with the gene encoding mNeonGreen, an mNeonGreen fragment was amplified by PCR with primers SalI-mNeonGreen-Fwd and mNeonGreen-TAA-XbaI-Rev (Table S1). The fragment obtained was digested with SalI and XbaI and ligated into SalI- and XbaI-digested pCD191 (25). The resulting plasmid was then transformed into *C. glutamicum* RES 167 to obtain RES 167 *divIVA*::*divIVA-mNeonGreen*.

### Construction of *C. glutamicum* RES 167 *pEKEX-divIVA*-Dendra2.

To construct a strain overexpressing DivIVA-Dendra2, the *divIVA* gene was introduced into the IPTG-inducible pEKEX-2 vector (49) with primers SalI RBS DivIVA forward and BamHI DivIVA reverse (Table S1). In frame with the *divIVA* gene we cloned the gene for *dendra2* with primers BamHI Dendra2 forward and SacI Dendra2 reverse (Table S1). The constructed plasmid was transformed into *C. glutamicum* RES 167, giving strain GGB1C9 (Table S1).

### qPCR.

Cell cultures (5 ml) were harvested during the exponential growth phase (OD_600_ of ~3.0), and cell pellets were stored at −80°C. Stored pellets were resuspended in 350 µl of RA1 buffer supplemented with 3.5 µl of β-mercaptoethanol and lysed with a FastPrep homogenizer. RNA was isolated with the NucleoSpin RNA kit (Macherey-Nagel) according to the manufacturer’s protocol. Synthesis of cDNA was performed with the RevertAid H Minus First Strand cDNA synthesis kit (Thermo Scientific) with RevertAid H Minus Moloney murine leukemia virus reverse transcriptase according to the manufacturer’s recommendations. qPCR analysis to determine *divIVA* transcript levels was run in biological triplicate with three housekeeping genes (*thrC*, *glnA*, and *gyrB*) as internal references. Primer efficiency assays were performed to control for primer binding. All qPCR experiments were performed with KAPA SYBR Fast QPCR MasterMix (Peqlab Biotechnologie GmbH).

### Azido-d-alanine staining.

Azido-d-alanine staining was performed as previously described (50). The compounds used were obtained from Jena Bioscience. Cultures of *C. glutamicum* or *C. glutamicum* DivIVA-mCherry were inoculated from overnight cultures to an OD_600_ of 0.2 and grown to an OD_600_ of 1 to 1.2. Next, 200-µl aliquots were transferred to the wells of a microtiter plate and further incubated in a ThermoMixer (Eppendorf) at 30°C and 850 rpm. Following 5 min of acclimatization, 1 µl of azido-d-alanine (1 M) (or 1 µl d-alanine [1 M] for the controls) was added and the mixture was incubated for 5 min (approximately 5% of the generation time). Cells were harvested, washed with phosphate-buffered saline (PBS, pH 7.4), and resuspended in 200 µl of PBS. Subsequently, 1 µl of DBCO-fluorophore (carboxyrhodamine 110 or Texas Red at 1 mM in dimethyl sulfoxide) was added and the mixture was incubated for 20 min at 30°C at 80 rpm in the dark. The cells were harvested and washed three times with PBS containing 0.1% Tween 20 to effectively remove the unbound fluorophore. Cells were then resuspended in PBS for microscopy. For dual labeling, PBS and PBS–0.1% Tween 20 were replaced with prewarmed BHI (30°C) following the first labeling.

### Live-dead staining.

Viable cell counts were performed with the Bacteria Live/Dead Staining kit (PromoKine) according to the manufacturer’s recommendations.

### Wide-field microscopy.

Wide-field microscopy was performed on a Zeiss Axio Observer Z1 microscope equipped with a Hamamatsu OrcaR2 camera and a Plan-Apochromat 100×/1.4 oil Ph3 objective (Zeiss). Fluorescence was visualized with the appropriate filter sets (Zeiss). An environmental chamber set to 30°C was used for time-lapse studies. Images were acquired with ZEN (Zeiss) and processed with Fiji (31). Final image assembly was conducted with Adobe Photoshop.

### Localization microscopy.

To prepare cells for PALM imaging, they where harvested and fixed with 1% formaldehyde in PBS (1.78 g ⋅ liter^−1^ Na_2_HPO_4_ ⋅ 2H_2_O, 1.42 g ⋅ liter^−1^ Na_2_HPO_4_, 8 g ⋅ liter^−1^ NaCl, 0.2 g ⋅ liter^−1^ KCl, 0.27 2 g ⋅ liter^−1^ KH_2_PO_4_ [pH 7.4]) for 20 min at 30°C on a shaker and washed three times in PBS-G (PBS containing 10 mM glycine) to stop the reaction.

Prior to use, the imaging chambers (eight wells; Nunc) were cleaned by subsequent rounds of sonication (20 min per step) with the following solutions: 0.1 M HCl, double-distilled H_2_O (ddH_2_O), 60% ethanol, and ddH_2_O. Subsequent to sonication, the chambers were dried and plasma cleaned (15 mA for 30 s in a Cressington 208 Carbon High Vacuum Carbon Coater). To facilitate attachment of the fixed cells, 0.1% poly-l-lysine (wt/vol) was added to the chambers and they were incubated for 1 h. The chambers were then washed three times with PBS-G, and 1.5 to 2 µl of a 1:1,000 dilution of TetraSpeck beads (Thermo Fisher) was homogeneously distributed over the glass surface (the optimal concentration will yield three to five beads per field of view). The chamber was consecutively filled with PBS-G and fixed cells (the optimal concentration depends on the conditions and organism). The chamber was centrifuged at 200 × *g* to facilitate cell sedimentation and attachment to the glass surface.

A Zeiss ElyraP1 was used for PALM imaging (Alpha Plan-Apochromat 100×/1.46 oil objective DIC M27). The 488-nm laser and 495- to 550-nm bandpass and 750-nm low-pass filters were used for both mNeonGreen and DBCO-carboxyrhodamine 110 imaging. For mNeonGreen, the parameters chosen were 50 ms with an electron-multiplying charge-coupled device (EMCCD) gain of 200 and 15% laser power. DBCO-carboxyrhodamine 110 localization was imaged by using 100 ms with an EMCCD gain of 50 and 20% laser power. Imaging was performed under a pseudo-total internal reflection fluorescence angle with a sample penetration of approximately 400 nm (63.97°) for all the conditions except EMB-treated RES 167 *divIVA*::*divIVA*-mNeonGreen; the background due to increased cell size and delocalized DivIVA made HiLo a more suitable approach (100 ms, an EMCCD gain of 200, 15% laser power, and 47.26°). The PALM images were calculated with the Zeiss ZenBlack software, and lateral drift was corrected by using the fluorescent beads as position references. Events originated by single molecules were then isolated by filtering for photon number (400 to 920). The resulting point spread function (PSF) distribution had a Gaussian shape, suggesting that it originated from a single population. Further filtering was used to eliminate outliers (PSF, 100 to 200 nm; precision, <50 nm).

### SEM.

Samples for SEM were dropped onto a glass slide covered with a coverslip, shock frozen with liquid nitrogen, and fixed with 2.5% glutaraldehyde in fixative buffer containing 25 mM cacodylate, 75 mM NaCl, and 2 mM MgCl_2_ (pH 7.0). The samples were washed with fixative buffer, postfixed with 1% OsO_4_ in fixative buffer, and washed three times with the fixative buffer, followed by ddH_2_O. The samples were dehydrated with increasing concentrations of acetone and then critical-point dried with liquid CO_2_. The samples were mounted on carbon stubs with conductive silver and sputtered with platinum (3 to 5 nm). High-resolution micrographs were taken with an Auriga scanning electron microscope (Zeiss).

### Transmission electron microscopy.

Samples for transmission electron microscopy were used to fill 3-mm aluminum sandwich carriers, frozen at a high pressure (200 MPa) with an HPM 100 (Leica), freeze substituted with 2% OsO_4_ and 0.2% uranyl acetate in acetone, and infiltrated with and embedded in Spurr’s epoxy resin (ScienceServices). Following polymerization, ultrathin sections were cut with a diamond knife with an UltraCut E ultra microtome (Reichert Jung). The ultrathin sections were poststained with lead acetate. Electron micrographs were taken with a Zeiss EM 912 transmission electron microscope with an integrated Omega Filter operated in the zero-loss state.

### AG preparation and analysis.

SDS- and proteinase K-purified cell walls were prepared as previously described (21). In brief, cells from 2-liter cultures were harvested in the early logarithmic phase and disrupted with glass beads. The crude cell walls obtained were purified by consecutive SDS and proteinase K treatments. The yield of pure cell walls normalized per OD unit was 1.3 times greater for EMB-treated cells. AG was released from cell walls by incubation in 0.05 M H_2_SO_4_ (50 mg of purified cell wall in 5 ml of H_2_SO_4_ for 4 days at 37°C). Extracted cell walls were pelleted (15 min at 3,000 × *g* and 20°C). Sulfuric acid was removed from the supernatant by precipitation with Ba(OH)_2_, and the supernatant was dried in a SpeedVac. Sugars were liberated by mild acid hydrolysis (2 M HCl, 3 h, 100°C) and determined and quantified by TLC performed on silica gel 60 plates (Merck) with two runs in an acetonitrile-water (85:15, vol/vol) solvent system (51). Sugar visualization was performed with diphenylamine-aniline (20 min 100°C). Signals were quantified with a Bio-Rad ChemiDoc System.

### MA extraction and analysis.

MA extraction was performed by a modified protocol previously described (52). MA methyl esters (MAMEs) were extracted by acidic methanolysis. A 10-mg cell wall sample was suspended in 600 µl of dry methanol–toluene–95% sulfuric acid (30:15:1, vol/vol/vol) in glass tubes with polytetrafluoroethylene-lined lids and incubated for 16 h at 50°C. After cooling to room temperature, 400 µl of petroleum ether (boiling point, 60 to 80°C) was added. Following mixing and phase separation, the petroleum ether phase containing the MAMEs was removed and dried under a nitrogen stream.

The dried sample was dissolved in 10 µl of petroleum ether (60 to 80°C), and 1 µl was used for TLC.

TLC was carried out on silica gel 60 plates (Merck). Plates were run once in toluene-acetone (97:3, vol/vol). MAMEs were detected with molybdatophosphoric acid (3% in isopropanol, 20 min, 100°C). Following development, the yellow background was bleached with ammonia vapor and the signals were quantified with the Bio-Rad ChemiDoc System. Since no standards for corynomycolates are available, MAME levels could only be relatively compared.

### Mycomembrane staining with MarinaBlue-DHPE.

Cells were dried in a small spot on a microscope slide in a stream of synthetic air and overlaid with 2 µl of DHPE-MarinaBlue (Life Technologies, Inc.) working solution (10 µg ⋅ ml^−1^ in methanol, freshly prepared from a 1-mg ⋅ ml^−1^ stock solution in ethanol-toluene [1:1, vol/vol]), and then dried again. Unbound dye was removed by rinsing with 500 µl of filtered sterile water. Following drying, 5 µl of bovine serum albumin (BSA; 10 mg ⋅ ml^−1^) was applied evenly over the cells and dried. For rehydration, the BSA-coated cells were covered with 1 µl of water and a large coverslip and analyzed by fluorescence microscopy with the 4',6-diamidino-2-phenylindole (DAPI) channel (excitation wavelength, 358 nm; emission wavelength, 463 nm).

### Lectin staining of PG with FITC-labeled WGA.

Cells were spread on a glass slide and dried as described for DHPE-MarinaBlue staining. Following heat fixation, 10 µl of FITC-WGA (1 mg ⋅ ml^−1^ in PBS [pH 7.4] containing 0.05% sodium azide; Sigma) was evenly distributed over the fixed cells, and they were incubated for 10 min in the dark. Next, the cells were rinsed three times with 500 µl of filtered sterile water and then dried. For fluorescence microscopy (green fluorescent protein channel; excitation wavelength, 489 nm; emission wavelength, 509 nm), cells were rehydrated with 1 µl of water and covered with a large coverslip.

For double staining of PG and mycomembrane, cells were first labeled with FITC-WGA and subsequently stained with DHPE-MarinaBlue.

### Detection of antibiotic interaction by checkerboard resazurin microtiter assay (REMA).

The antibiotic interaction assay was performed as described before (53). For *C. glutamicum*, 2-fold serial dilutions of EMB starting at the MIC were prepared in 96-well microtiter plates in vertical or horizontal rows (in four serial dilutions each; 100 µl, calculated for 120 µl). The antibiotics PEN, CARB, or RIF were added in the opposite direction (1.2-µl volumes of 100-fold stock solutions), starting at the respective MICs. The first vertical and horizontal rows served as negative controls.

An exponential-phase *C. glutamicum* culture was diluted to an OD_600_ of 0.001, and 20 µl was added to each well. The microtiter plate was incubated in a ThermoMixer at 30°C and 700 rpm for 16 h. A 20-µl volume of resazurin (0.01% in distilled water, filter sterilized) was then added to each well, and the plate was incubated in a ThermoMixer at 30°C and 300 rpm for 30 min. Tests were done in parallel, both in a clear microtiter plate for visual inspection (no growth = no turnover of resazurin [blue]; growth/viability = turnover to resorufin [pink]) and in a black microtiter plate for fluorescence measurement. Fluorescence of resorufin was recorded in a Tecan Infinite M200 Pro (Tecan) microplate reader (excitation wavelength, 560 nm; emission wavelength, 590 nm). Mean values calculated from two to five experiments are shown in a heat map prepared with R.

For *M. phlei*, the checkerboard REMA was performed as described above, in BHI in 48-well plates in a total volume of 250 µl. An exponential-phase culture of *M. phlei* (grown in DSMZ medium no. 219) was allowed to settle, and the supernatant with suspended cells without aggregates was used for inoculation. This supernatant was diluted in BHI to an OD_600_ of 0.04, and 20 µl was added to each well. Plates were covered with Breathe-Easy sealing membrane (Sigma-Aldrich) and incubated in an orbital shaker at 37°C and 250 rpm for 48 h. After removal of the sealing membrane, 20 µl of resazurin (0.02% in distilled water, filter sterilized) was added to each well. Plates were shaken for 30 min at 37°C and 80 rpm. After a picture was taken, 120 µl was transferred from each well into a black microtiter plate and fluorescence was recorded as described above. Heat maps were created as described above.

### Computational image analysis.

For the acquisition of data from the multichannel fluorescent images, a plug-in for the open-source image analysis platform Fiji was created (31). The algorithm provides the possibility to analyze numerous images in an adequate timespan. The first step is the creation of a binary image from the phase-contrast channel by using the implemented function AutoThreshold (default mode). Next, the outline of each cell is automatically detected with the wand tool, yielding regions of interest covering all of the channels present. The algorithm developed uses the maximal distance of each outline to determine the centerlines of single cells. Subsequently, perpendicular lines reaching toward the cell outline are constructed along these centerlines. By determining the center of the perpendicular lines, a more accurate centerline is created, reflecting the cell’s actual curvature and length. A data set is created for each cell containing the length and the width along the length (in micrometers), as well as the linear fluorescence profiles of the centerlines and the corresponding perpendicular lines. Evaluation and visualization of the data were performed with the statistical open-source software R containing the default packages Hmisc and colorspace. The algorithm developed was used to sort the cells by length and to translate the numeric data of the fluorescence profiles into a color code, providing channel-specific heat maps of the entire experiment obtained by aligning the centerline profiles, as well as heat maps of single cells created by aligning the profiles of the perpendicular lines. Videos were created by using the open-source software solutions FFmpeg and ImageMagick.
