# Supplementary figures and images for: The Antituberculosis Drug Ethambutol Selectively Blocks Apical Growth in CMN Group Bacteria
Source: mBio. 2017 Feb 7;8(1):e02213-16. doi: 10.1128/mBio.02213-16 (PMC5296602; doi:10.1128/mBio.02213-16)

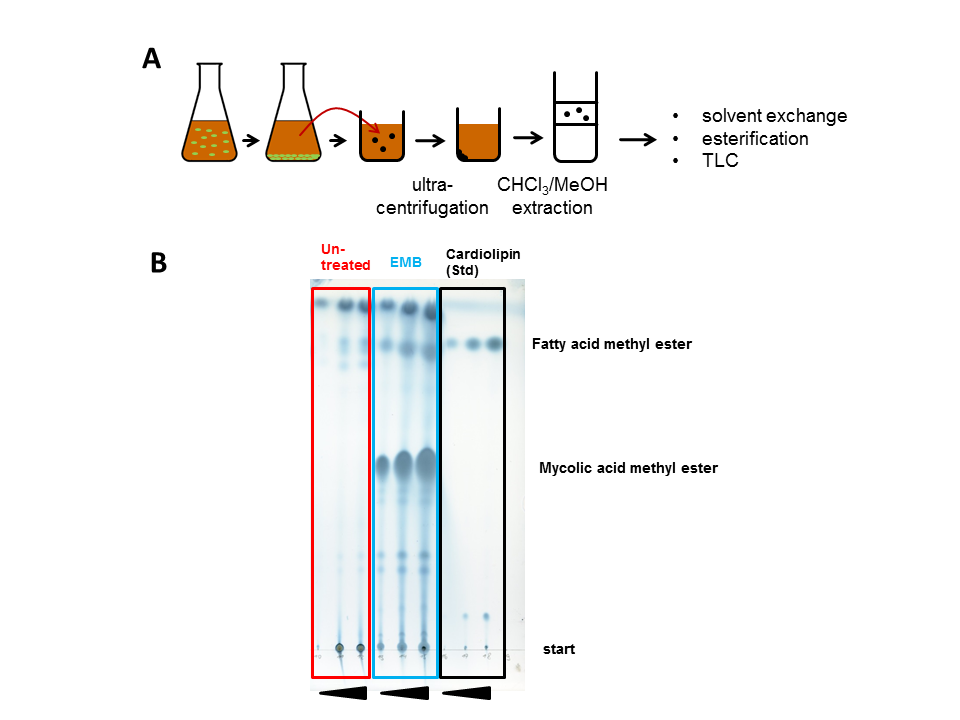

Supplement: FIG S1 [file mbo001173175sf1.tif]

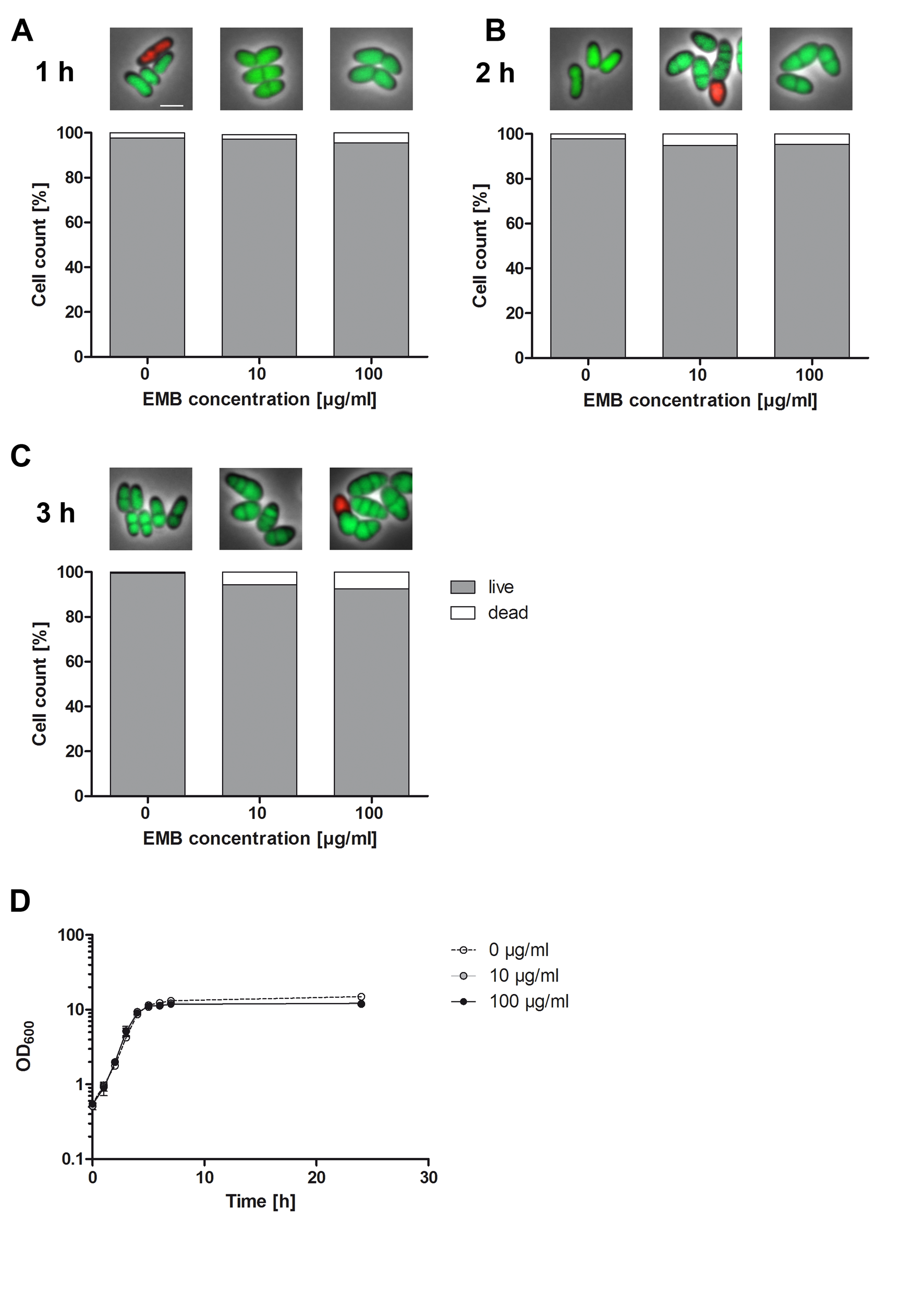

Supplement: FIG S2 [file mbo001173175sf2.tif]

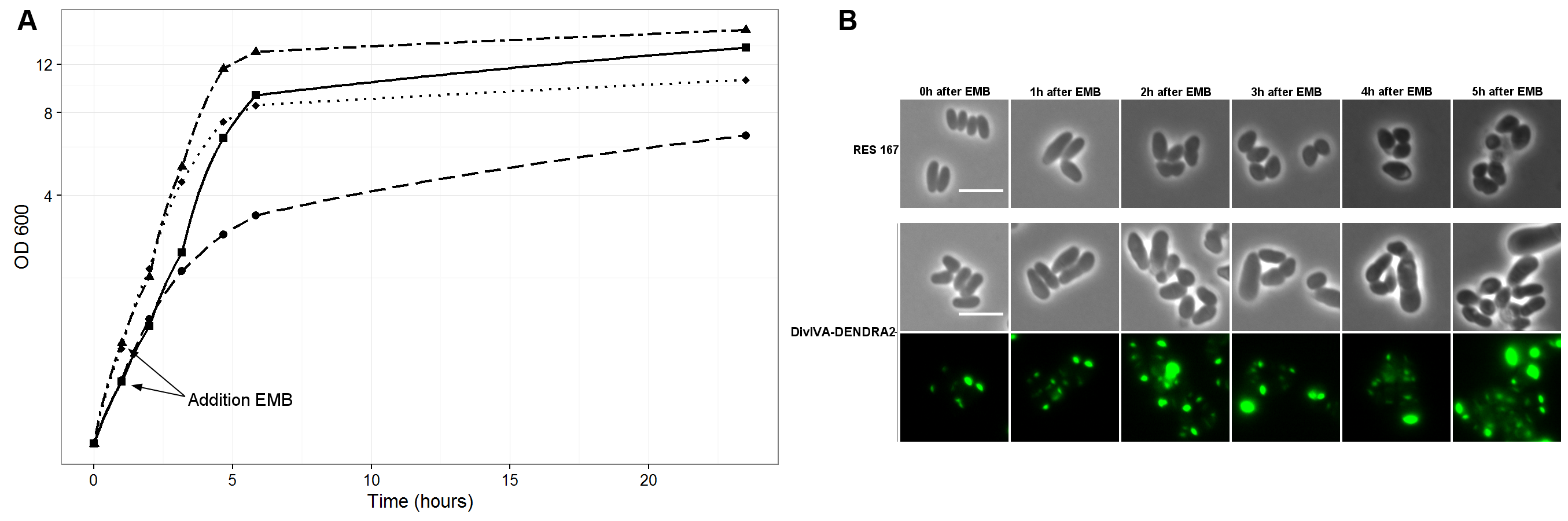

Supplement: FIG S3 [file mbo001173175sf3.tif]

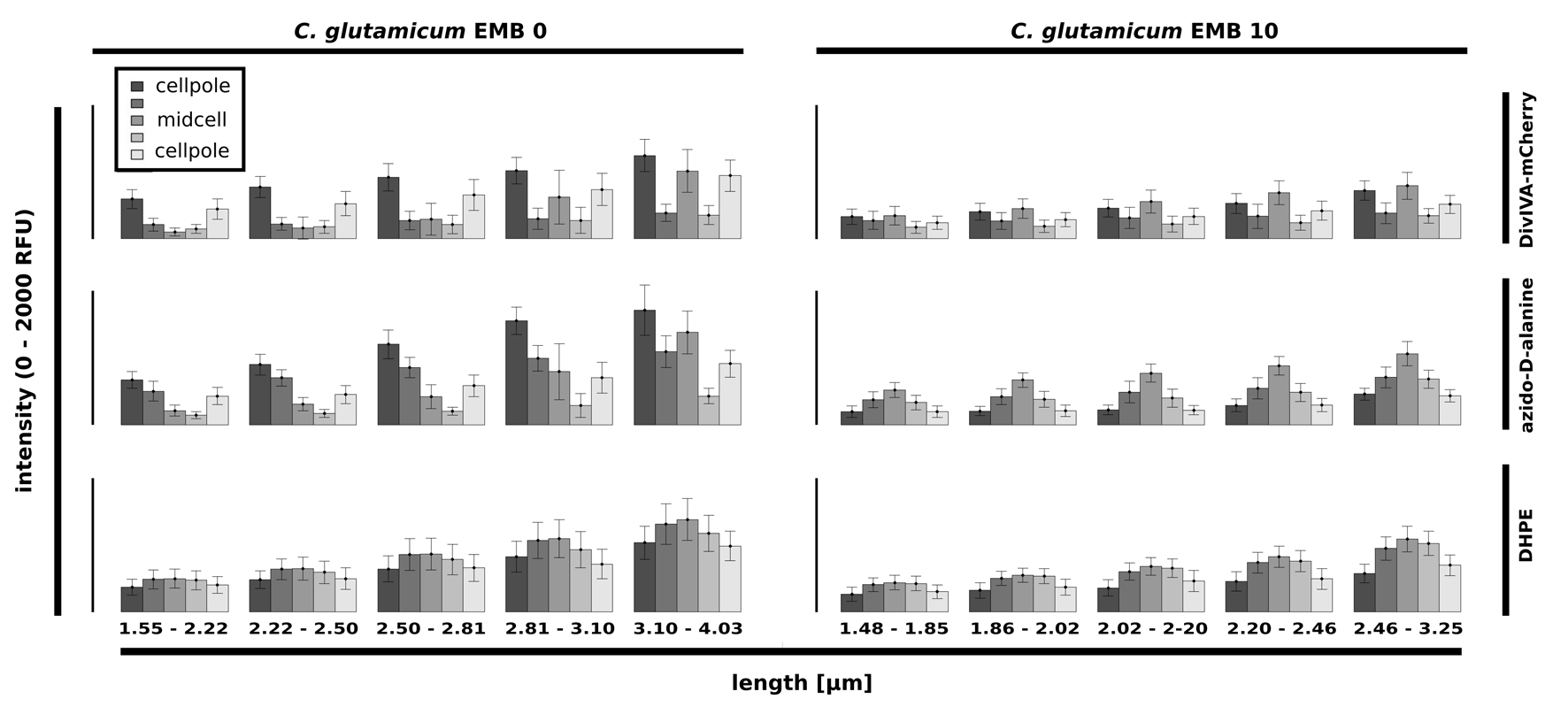

Supplement: FIG S4 [file mbo001173175sf4.tif]

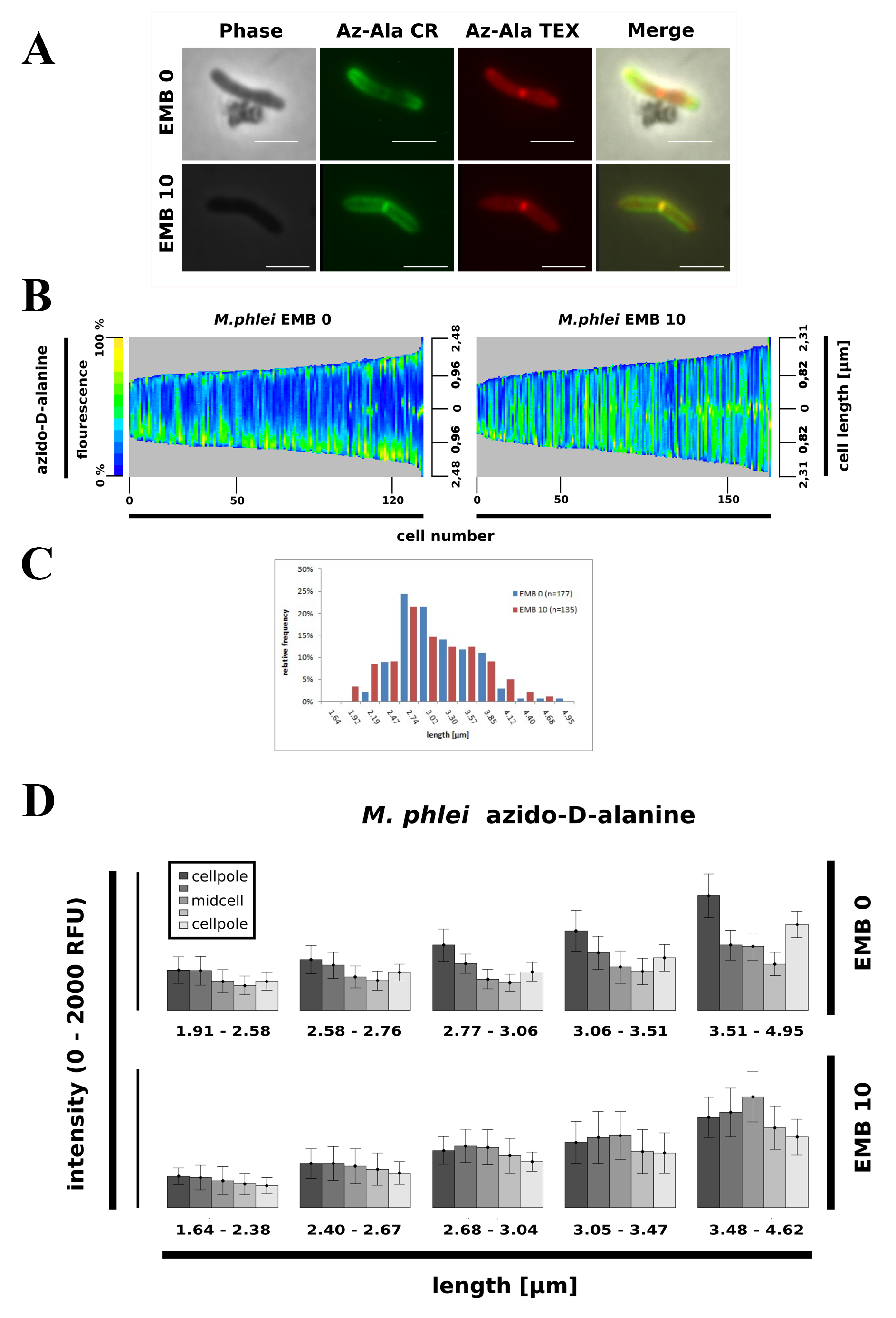

Supplement: FIG S5 [file mbo001173175sf5.tif]

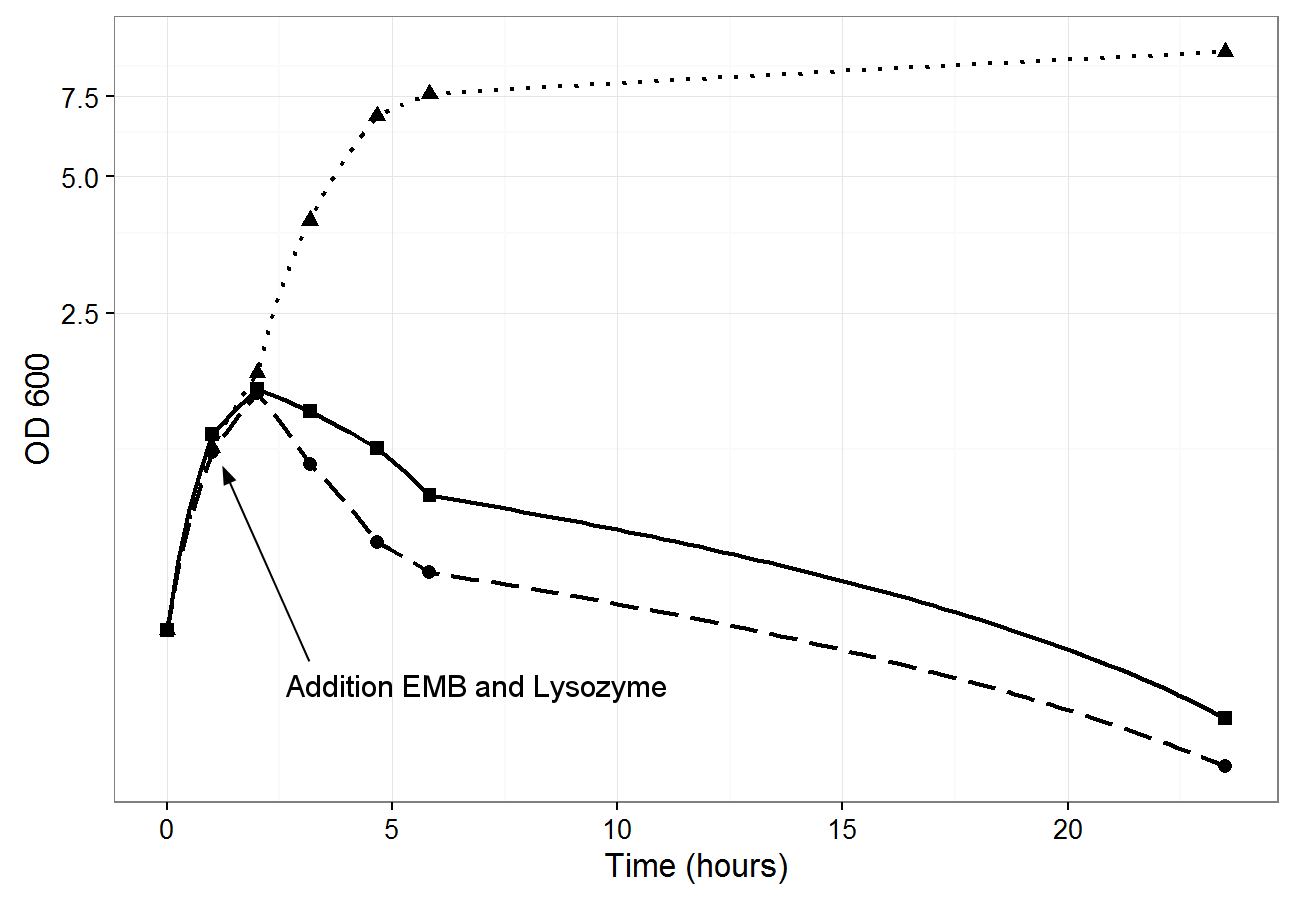

Supplement: FIG S6 [file mbo001173175sf6.tif]

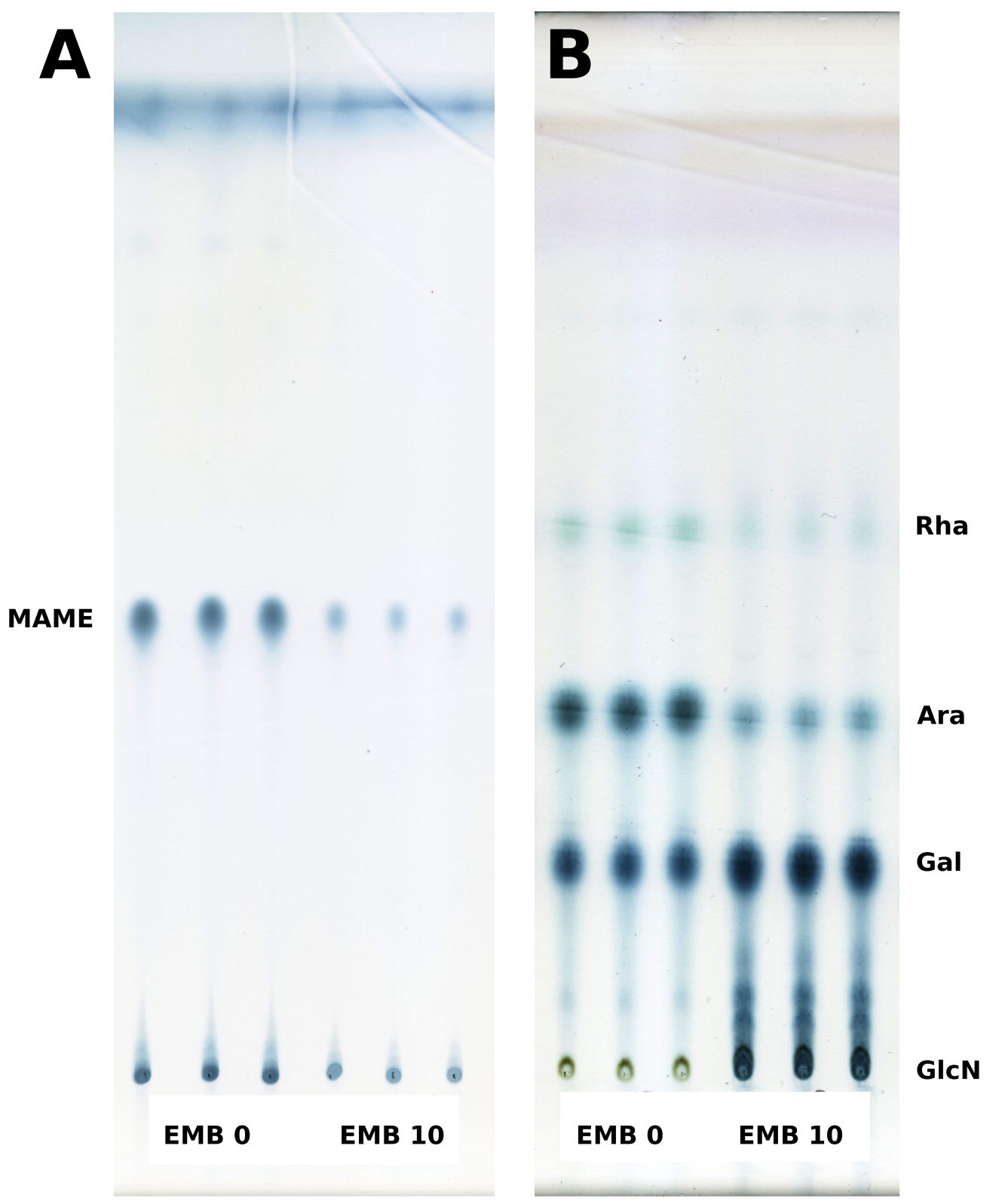

Supplement: FIG S7 [file mbo001173175sf7.tif]
