# Supplementary material for: The Antituberculosis Drug Ethambutol Selectively Blocks Apical Growth in CMN Group Bacteria
Source: mBio. 2017 Feb 7;8(1):e02213-16. doi: 10.1128/mBio.02213-16 (PMC5296602; doi:10.1128/mBio.02213-16)
Supplement: TABLE S1 [file mbo001173175st1.docx]

**Table S1: Strains and oligonucleotides used in this study**

| **Name** | **Genotype/Description** | | **Reference** |
| --- | --- | --- | --- |
| ***E. coli*** |  | |  |
| DH5α | F^-^ φ80*lac*Z.M15(*lac*ZYA-*arg*F)U169 *rec*A1 *end*A1 *hsd*R17(r_k_-,m_k_+) *pho*A *sup*E44 *thi*-1 *gyr*A96 *rel*A1 λ^−^ | | Invitrogen |
| ***C. glutamicum*** |  | |  |
| ATCC 13032 | Type strain | | Laboratory stock |
| Res 167 | Restriction deficient *C. glutamicum* | | (51) |
| CDC010 | Res 167, DivIVA-mCherry | | (25) |
| BSC025 | Res 167, FtsW-CFP | | (21) |
| BSC026 | Res 167, RodA-YFP | | (21) |
| GGCB1C8 | Res 167, DivIVA-mNeonGreen | | This study |
| GGB1C9 | Res 167, pEKEX-DivIVA-Dendra2 | | This study |
| ***M. phlei*** |  | |  |
| **43239** | **Type strain** | | DSM |
| **Oligonucleotides** | | | |
| **Name** | | **Sequence** |  |
| ***qPCR primer*** | |  | |
| qDivIVA F | | CATCCTCCAGCACCACTAAG | |
| qDivIVA R | | CATGTGGGTGTCCACATTTG | |
| qGlnA F | | GCGGACAGCAGGAAATCAAC | |
| qGlnA R | | CAGTGGCTTAGGCATGAAGG | |
| qGyrB F | | GTCCACACCTTCGCCAACAC | |
| qGyrB R | | TTGGTCTGGCCTTCGAACTG | |
| qThrC F | | GCCGTGAACTCCATCAACTG | |
| qThrC R | | CTGCGCAAATGTCACCGAAG | |
| qDivIVA F | | CATCCTCCAGCACCACTAAG | |
| qDivIVA R | | CATGTGGGTGTCCACATTTG | |
| ***divIVA-mNeonGreen*** | |  | |
| SalI-mNeonGreen-Fwd | | TATGTCGACATGGTGAGCAAGG | |
| mNeongreen-TAA-XbaI-Rev | | ATGTCTAGATTA CTTGTACAGCTCGTC | |
| ***divIVA-Dendra2*** | |  | |
| SalI RBS DivIVA forward: | | CATGTCGACATCGAAGGGAATCCGCAA ATGCCGTTGACTCCAG | |
| BamHI DivIVA reverse | | CCGGGATCCCTCACCAGATGGC | |
| BamHI Dendra2 forward | | GAGGGATCCATGAACACCCCG | |
| SacI Dendra2 reverse | | CCGGAGCTCTTACCACACCTGG | |

* Restriction sites are underlined
